# The metabolic syndrome-cancer axis: global research trends and clinical landscapes

**DOI:** 10.3389/fendo.2026.1799202

**Published:** 2026-06-22

**Authors:** Dianzhe Tian, Xinshi Li, Jiajie Lin, Zuyi Yang, Xinyu Zhao, Zixuan Hu, Haitao Zhao, Shunda Du, Shengzhi Liu, Lei Zhang, Yiyao Xu, Xin Lu

**Affiliations:** 1 Department of Liver Surgery, State Key Laboratory of Complex Severe and Rare Diseases, Peking Union Medical College Hospital, Chinese Academy of Medical Sciences and Peking Union Medical College, Beijing, China; 2 Eight-year Medical Doctor Program, Chinese Academy of Medical Sciences and Peking Union Medical College, Beijing, China; 3 Department of Pharmacology, College of Pharmaceutical Sciences of Capital Medical University, Beijing, China; 4 Key Laboratory of Ocular Fundus Disease, Chinese Academy of Medical Sciences, Beijing, China; 5 Department of Ophthalmology, Peking Union Medical College Hospital, Beijing, China; 6 Beijing Area Major Laboratory of Peptide and Small Molecular Drugs, Engineering Research Center of Endogenous Prophylactic of the Ministry of Education of China, Beijing Laboratory of Biomedical Materials, Beijing, China

**Keywords:** bibliometric analysis, biomarker, cancer, metabolic syndrome, metabolism

## Abstract

**Background:**

The link between metabolic syndrome (MetS) and cancer has been widely studied, but overall research patterns, shared mechanisms, and applications remain unclear. A bibliometric and bioinformatics analysis is needed to identify trends and clarify the molecular pathways connecting MetS to cancer.

**Methods:**

A bibliometric analysis using Web of Science and PubMed assessed publication trends, collaboration, key authors/journals, and research topics from 2000 to 2024. Visualization and analysis used VOSviewer, CiteSpace, and Bibliometrix. Also included bioinformatics for protein interaction networks, hub genes, and enrichment analysis via R pipelines.

**Results:**

Research output grew mainly driven by China and the US, with Europe showing high collaborative centrality. Thematic analysis shifted from isolated metabolic issues to integrated MetS phenotypes, cancer prognosis, and interventions. Bioinformatics found hub genes and pathways linking metabolic dysregulation with tumor development, especially involving inflammation, insulin signaling, and metabolic reprogramming.

**Conclusions:**

This analysis supports a bidirectional MetS-cancer link and shifts focus from tumor-centric to a holistic view of the tumor-host metabolic system. Targeting metabolic dysfunction may improve cancer prognosis, treatment tolerance, and outcomes.

## 1 Introduction

Metabolic syndrome (MetS) comprises a complex of interrelated risk factors that increase the risk of cardiovascular disease and diabetes. These factors include dysglycemia, elevated blood pressure, elevated triglyceride levels, reduced high-density lipoprotein cholesterol levels, and abdominal obesity (1–3). The National Cholesterol Education Program–Third Adult Treatment Panel (NCEP ATP III) formalized a practical diagnostic framework in 2001, defining MetS by the presence of any three of five routinely measured parameters, with waist circumference (WC) as a key metric of central adiposity (1). In 2005, the International Diabetes Federation (IDF) proposed a modified definition requiring abdominal obesity and incorporating sex- and ethnicity-specific WC thresholds (2). In 2009, harmonized diagnostic criteria were established through an international consensus effort, providing a unified framework for global application (3). In 2023, the American Heart Association (AHA) expanded the conceptual framework by introducing the cardiovascular-kidney-metabolic (CKM) syndrome, reflecting the interconnected metabolic, cardiovascular and renal pathways that contribute to shared disease progression (4).

The epidemiology of MetS demonstrates a substantial and growing global burden, with marked regional variability shaped by demographic, lifestyle and socioeconomic determinants (5). In North America and Europe, prevalence estimates from large population-based cohorts range from approximately 25% to more than 40%, driven largely by rising obesity and dysglycemia (6–8). In Asia, prevalence remains highly heterogeneous, reflecting regional differences in dietary transitions, physical activity patterns, genetic predisposition, and diagnostic thresholds (9–11). Data from Africa similarly suggest increasing rates of MetS, particularly among populations affected by HIV (12). Although standardized criteria for children and adolescents remain lacking, emerging evidence indicates that MetS is becoming more common in younger individuals in parallel with increasing rates of obesity and sedentary behavior (13). Collectively, these patterns highlight the widespread and multifactorial nature of MetS and underscore the need for early detection and region-specific preventive strategies.

Beyond its established cardiometabolic consequences, MetS has gained increasing recognition as a significant determinant of cancer risk and progression. Meta-analyses demonstrate that MetS increases the incidence of several cancers, including colorectal, liver, endometrial, and pancreatic cancers (14). MetS promotes a pro-tumorigenic environment through chronic inflammation, insulin resistance and IGF-1 axis activation, adipokine dysregulation, and metabolic reprogramming, thereby facilitating tumor initiation and metastatic dissemination. Recent findings further reveal that MetS-induced metabolic alterations can reshape the tumor microenvironment, notably enhancing the propensity for colorectal cancer to metastasize to the liver (15, 16). Clinically, MetS is associated with poorer oncologic outcomes, including higher recurrence rates, diminished therapeutic responsiveness, and reduced survival (17). Notably, evidence from endometrial cancer demonstrates that targeted correction of MetS components, particularly weight reduction and improved insulin sensitivity, can enhance treatment efficacy, underscoring its potential as a modifiable prognostic factor (18). Despite extensive mechanistic and clinical investigations, current research on MetS–cancer comorbidity remains fragmented and lacks an integrated, quantitatively informed framework.

Key knowledge gaps in MetS research include the evolution of conceptual frameworks, geographic disparities, and the disconnect between mechanistic studies and clinical applications. A systematic bibliometric approach is necessary to map these gaps, utilizing publication data, citation networks, and collaboration dynamics to identify trends and emerging hotspots (19, 20). This approach provides a more efficient and objective means of navigating the literature, thereby overcoming the limitations of narrative reviews. Although studies have reported associations between cancer and MetS, an investigation that fully integrates these two fields remains lacking. Using bibliometric and bioinformatic methods, the study systematically delineates the knowledge landscape of MetS-cancer research over the past decade. Through in-depth analysis, we elucidate the developmental trajectories and emerging trends in this field, offering a conceptual framework to guide future academic exploration and to facilitate the translation of related findings into clinical practice (21).

## 2 Materials and methods

### 2.1 Data source

Relevant articles were retrieved from the Web of Science Core Collection (WoSCC), restricted to the Science Citation Index Expanded (SCI-EXPANDED) edition. The search strategy was developed collaboratively by all authors, in consultation with senior literature search experts (XL and YYX), using MetS-related and cancer-related search terms. The study selection process was conducted in two stages. An initial screening was performed based on article titles, followed by a secondary screening using abstracts. To identify cutting-edge advancements in clinical trials, we concurrently expanded our retrieval scope to include the latest clinical trials in PubMed. This was followed by an analysis of the retrieved data. After excluding unrelated studies (n = 8), we identified 17 clinical trials. The detailed search query is shown in **Table 1**. All data sources were searched on October 22, 2025. .

**Table 1:** PRISMA-based search strategy and study selection for Web of Science Core Collection (WoSCC) and PubMed.

PRISMA stage	WoSCC (bibliometric analysis)	PubMed (clinical trials)
Identification	Search strategy: TI = (#1) AND TI = (#2) #1: Cancer-related terms #2: MetS-related terms Initial records identified: n = 714 Limits: Article or Review, English, 2000 - 2024	Search strategy: (#1) AND (#2) Initial records identified: n = 25 Filter: *Clinical Trial*
Manual Screening	Excluded unrelated to MetS: n = 21 Excluded unrelated to cancer: n = 10 Duplicates removed: n = 3 Retracted articles: n = 2 Records without full metadata or citation data: n = 2	Excluded unrelated to MetS: n = 5; Excluded unrelated to cancer: n = 3
Eligibility	Eligible studies: n = 676	Eligible studies: n = 17
Included	Included in final analysis: n = 676	Included in final analysis: n = 17

Cancer-related terms (#1), “Tumors” OR “Neoplasia” OR “Neoplasias” OR “Neoplasm” OR “Tumor” OR “Cancer” OR “Cancers” OR “Malignant Neoplasm” OR “Malignancy” OR “Malignancies” OR “Malignant Neoplasms” OR “Neoplasm, Malignant” OR “Neoplasms, Malignant” OR “Benign Neoplasms” OR “Neoplasms, Benign” OR “Neoplasm, Benign” OR “Benign Neoplasm” OR “Hematologic Neoplasms” OR “Hematologic Malignancies” OR “Blood Neoplasms” OR “Blood Cancer” OR “Blood Cancers” OR “Leukemia” OR “Leukemias” OR “Lymphoma” OR “Lymphomas” OR “Myeloma” OR “Multiple Myeloma” OR “Hodgkin Disease” OR “Non-Hodgkin Lymphoma” OR “Acute Lymphoblastic Leukemia” OR “Chronic Lymphocytic Leukemia” OR “Acute Myeloid Leukemia” OR “Chronic Myeloid Leukemia” OR “Oncology”, MetS-related terms (#2), “metabolic syndrome” OR “Metabolic Syndromes” OR “Syndrome, Metabolic” OR “Syndromes, Metabolic” OR “Metabolic Syndrome X” OR “Insulin Resistance Syndrome X” OR “Syndrome X, Metabolic” OR “Syndrome X, Insulin Resistance” OR “Metabolic X Syndrome” OR “Syndrome, Metabolic X” OR “X Syndrome, Metabolic” OR “Dysmetabolic Syndrome X” OR “Syndrome X, Dysmetabolic” OR “Reaven Syndrome X” OR “Syndrome X, Reaven” OR “Metabolic Cardiovascular Syndrome” OR “Cardiovascular Syndrome, Metabolic” OR “Cardiovascular Syndromes, Metabolic” OR “Syndrome, Metabolic Cardiovascular” OR “Cardiometabolic Syndrome” OR “Cardiometabolic Syndromes” OR “Syndrome, Cardiometabolic” OR “Syndromes, Cardiometabolic”.

### 2.2 Data analysis

A analytical workflow integrating bibliometric assessment, network biology, and enrichment analysis was implemented across multiple computational platforms in this study. Bibliometric analyses were conducted using R (version 4.2.2), VOSviewer (version 1.6.20), and CiteSpace (version 6.4.R1) (22–25). The Bibliometrix R package (version 4.1.4) was applied to quantify collaborative interactions among countries (26). Publication output, citation counts, and keyword occurrence frequencies were extracted using VOSviewer. Keywords with similar meanings were consolidated to reduce redundancy. CiteSpace was employed to detect keywords exhibiting significant citation bursts within defined temporal intervals. International collaboration networks were visualized using an online bibliometric tool (https://bibliometric.com/). The counting method for VOSviewer was based on full counting. A synonym thesaurus file was applied to merge institutional names and keyword synonyms. CiteSpace was used with a time slicing interval of 1 year per slice. The selection criteria were set to Top N = 50 for keyword analysis and g-index (k = 25) for co-citation analysis. The Pathfinder algorithm was applied as the pruning strategy for both sliced networks and merged networks. Burst detection was performed using the Kleinberg algorithm, with the minimum duration set to 1 year.

For bioinformatics analysis, genes associated with MetS and cancer were obtained from the GeneCards database (https://www.genecards.org/) using the keywords “metabolic syndrome” and “cancer”, with a relevance score threshold of 10 or higher applied to filter results. Protein-protein interaction (PPI) networks were constructed via the STRING database (https://string-db.org) under a stringent confidence score cutoff of 0.9 (27). The resulting interaction networks were imported into Cytoscape (version 3.9.1) for further visualization. Hub genes were identified using centrality metrics from six cytoHubba algorithms: Betweenness, Closeness, Degree, Maximum Clique Centrality (MCC), and BottleNeck. Functional characterization was conducted using Gene Ontology (GO) enrichment in Biological Process (BP), Molecular Function (MF), and Cellular Component (CC), as well as Kyoto Encyclopedia of Genes and Genomes (KEGG) pathway analysis (28). Statistical significance was determined using hypergeometric testing, with false discovery rate control implemented through the Benjamini-Hochberg (BH) method (adjusted p-value < 0.05). Disease ontology (DO) enrichment analysis was conducted using the DOSE package (version 3.26.0) and the Jaccard index to quantify semantic similarity between disease terms. Hierarchical clustering using Ward’s minimum-variance method was used to group related disease categories. Redundant terms were filtered out using a tree height threshold of 0.85 to enhance interpretability while maintaining vital biological information.

**Figure 1:**
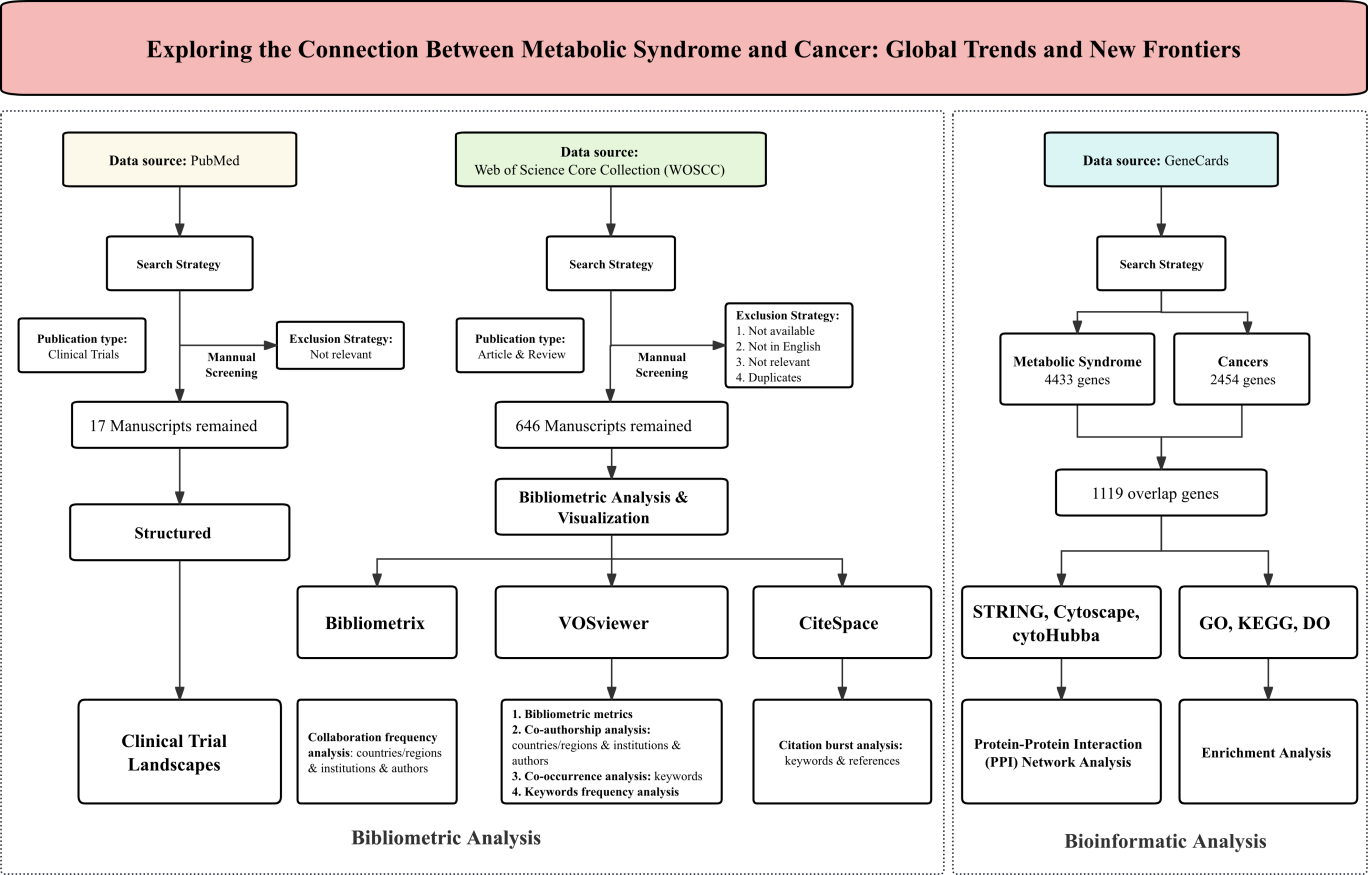
An overview of the flowchart of the study. Flowchart of the research study on metabolic syndrome in cancer, utilizing bibliometric and bioinformatic analyses. GO, Gene Ontology; KEGG, Kyoto Encyclopedia of Genes and Genomes; DO, Disease Ontology.

All computational procedures were carried out in R (version 4.2.2) under standardized conditions to guarantee reproducibility. The access date for all databases used in enrichment analysis was October 29, 2025. A schematic overview of the entire analytical workflow is shown in **Figure 1**.

## 3 Results

### 3.1 Overview of publication status

**Figure 2:**
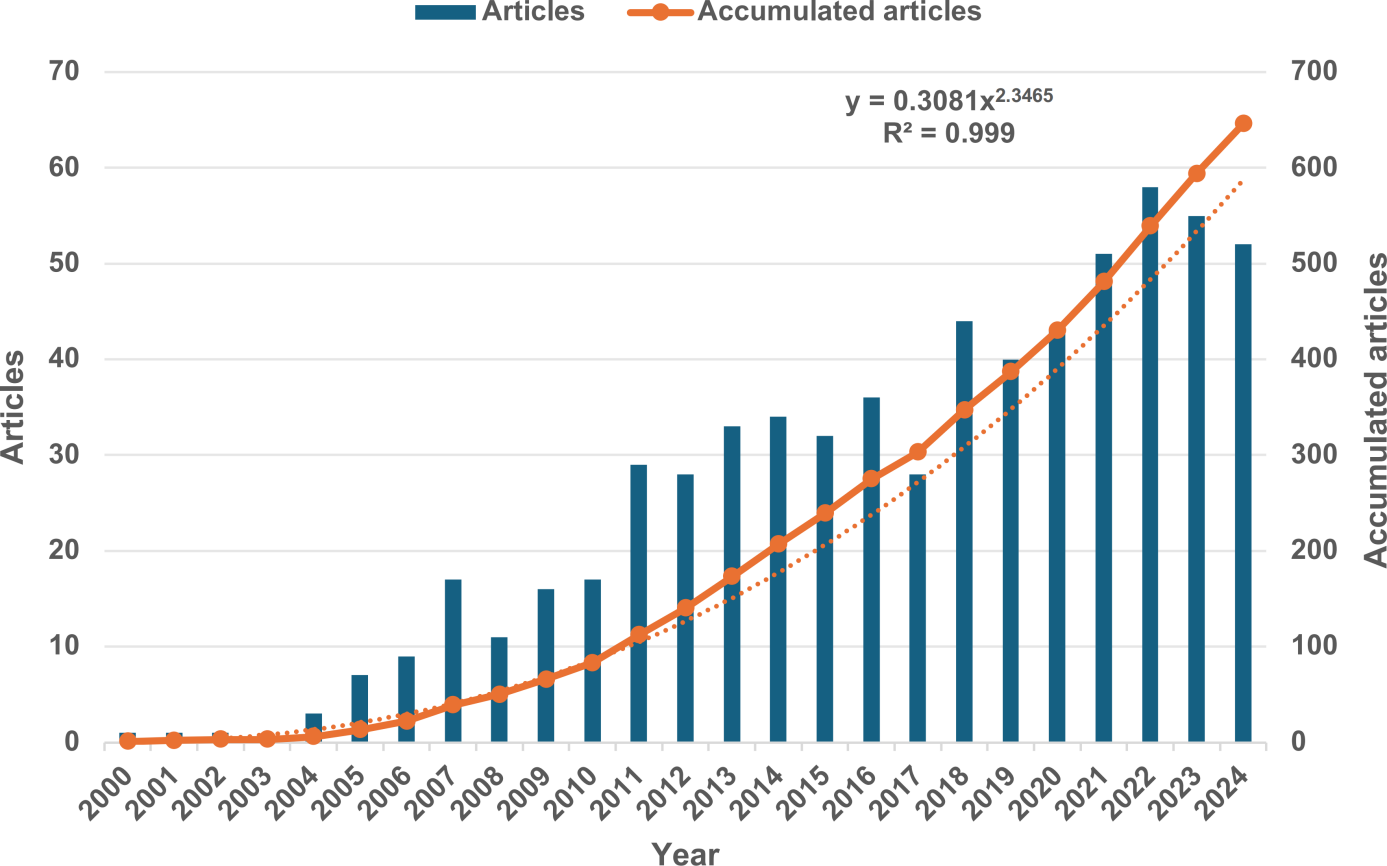
The number of publications per year and the cumulative number. The solid line represents the original data points of cumulative publication counts. In contrast, the dashed line represents the fitted curve based on a power function model (*y = 0.3081x^2.3465, R² = 0.999*, where y is the accumulated number of articles published and x is the year of publication).

This study included 676 conventional articles, as shown in **Figures 1**, **2** summarizes the annual and cumulative publication counts related to MetS in cancer. The number of publications began rising steadily and peaked in 2022 (N = 58). The relationship between cumulative publications and publication year was evaluated using a power function model, which accurately fit the trend (R^2^ = 0.999).

**Figure 3:**
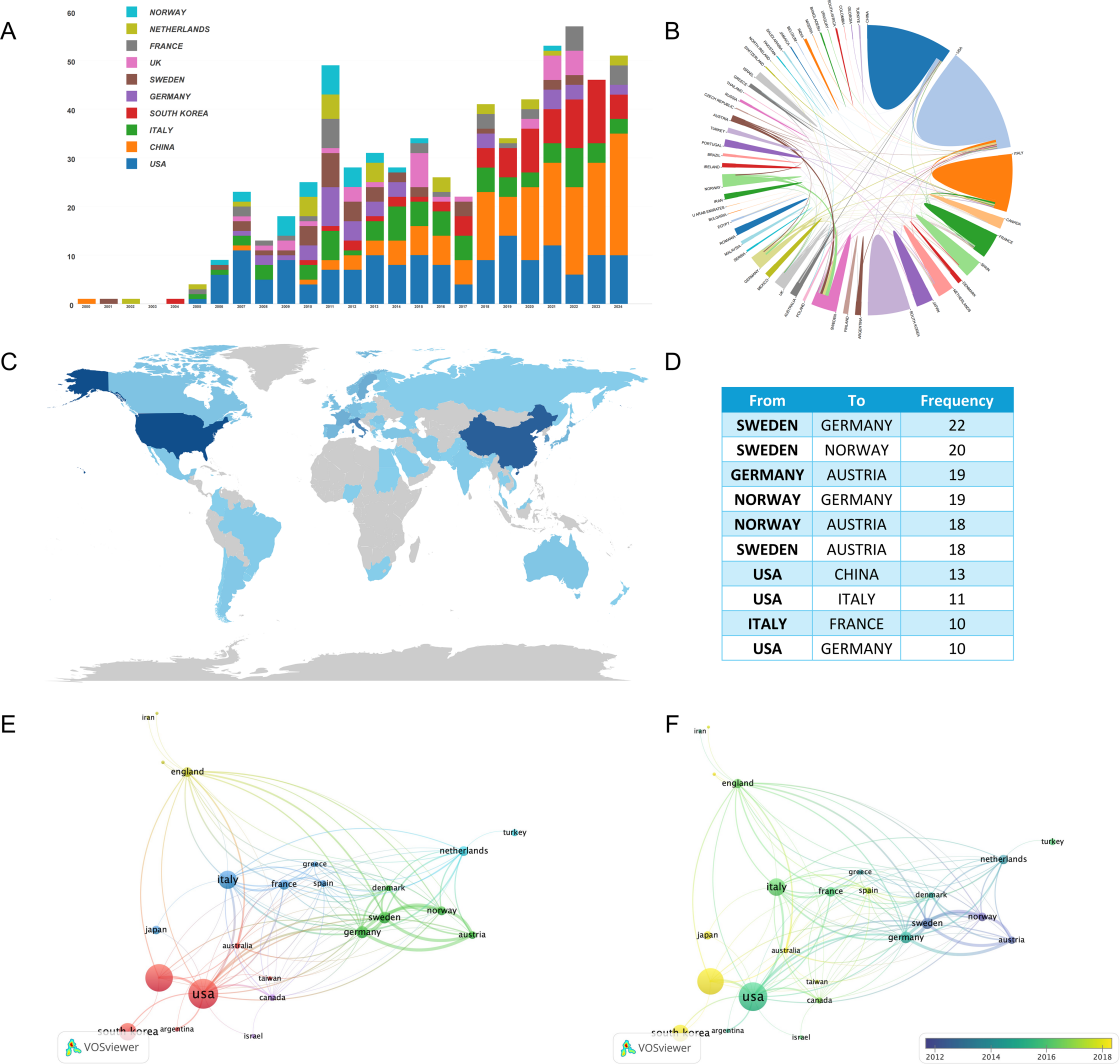
The individualized involvement of each nation in the domain of MetS in cancer. **(A)** The ratio of publications of the top 10 countries each year; **(B)** The network map of countries’ cooperation; **(C)** The geographical visualization of the national publication; **(D)**. Cooperation frequency of the top 16 countries/regions; **(E)** Clustering network of international co-authorship; **(F)** Time-overlapping network of international co-authorship.

Our bibliometric analysis of national/regional contributions revealed distinct geographical patterns in the productivity of research on MetS and cancer. As shown in **Table 2**, the top three countries by total citation count in terms of publication contribution are the United States(USA), Italy, and China. As illustrated in **Figure 3A**, China and the USA accounted for the most scholarly output, with China producing 144 publications compared to 118 from the USA, significantly surpassing South Korea (N = 68) and Italy (N = 66). The remaining countries/regions each contributed fewer than 25 publications. Longitudinal analysis indicated that the USA had maintained a consistent and significant contribution in this field since 2005. However, a pivotal shift occurred in 2018, when China’s annual publication output exceeded that of the USA. Although a minor rebound was observed in the USA the following year, China subsequently established sustained leadership in the years that followed. While the USA was an early pioneer in MetS–cancer research, Chinese researchers entered the field more recently and have made outstanding contributions in recent years. Geospatial visualization (**Figure 3C**), with darker colors denoting higher publication volumes, further mapped the global contribution landscape, confirming the central roles of China and the USA in terms of research productivity. Our network analysis (**Figures 3B, D**) indicated that Sweden was the most frequent collaborator, maintaining the closest cooperative ties with Germany (22 collaborations) and Norway (20 collaborations). Collaborative activities among other countries/regions were also notably active. After excluding countries that could not form collaborative clusters with others, we analyzed co-authorship patterns among the 24 countries that had published more than 5 relevant publications to characterize international research collaboration. In both the clustering network and the time-overlapping network, the size of each circle represents the volume of publications. In the clustering network, specifically, the color of each circle reflects the intensity of collaborative links among different research groups. The time-overlapping network indicates the mean publication year for each country within the research field. As depicted in **Figure 3E**, the 22 countries (2 excluded for not forming a cluster) under analysis are categorized into five distinct clusters, among which the largest one, marked in red, consists of six nations. **Figure 3F** also demonstrates that the USA initiated research efforts in MetS-associated cancer earlier, while contributions from Chinese researchers have only become prominent in recent years.

**Table 2:** Top 12 countries that contributed to publications in metabolic syndrome-cancer research, ranked by total citations.

Rank	Country	TC	Average TC	Articles	Articles %	SCP	MCP	MCP %
1	USA	7673	65.00	118	18.3	100	18	15.3
2	Italy	4598	69.70	66	10.2	45	21	31.8
3	China	2346	16.30	144	22.3	125	19	13.2
4	Sweden	1358	75.40	18	2.8	6	12	66.7
5	Korea	1108	16.30	68	10.5	63	5	7.4
6	Japan	979	44.50	22	3.4	20	2	9.1
7	Netherlands	927	54.50	17	2.6	15	2	11.8
8	Germany	868	62.00	14	2.2	5	9	64.3
9	Spain	845	70.40	12	1.9	8	4	33.3
10	Norway	544	90.70	6	0.9	4	2	33.3
11	France	517	34.50	15	2.3	11	4	26.7
12	UK	499	33.30	15	2.3	6	9	60

TC, total citation; SCP, single country publications; MCP, multiple country publications; USA, the United State; UK, the United Kingdom.

**Figure 4:**
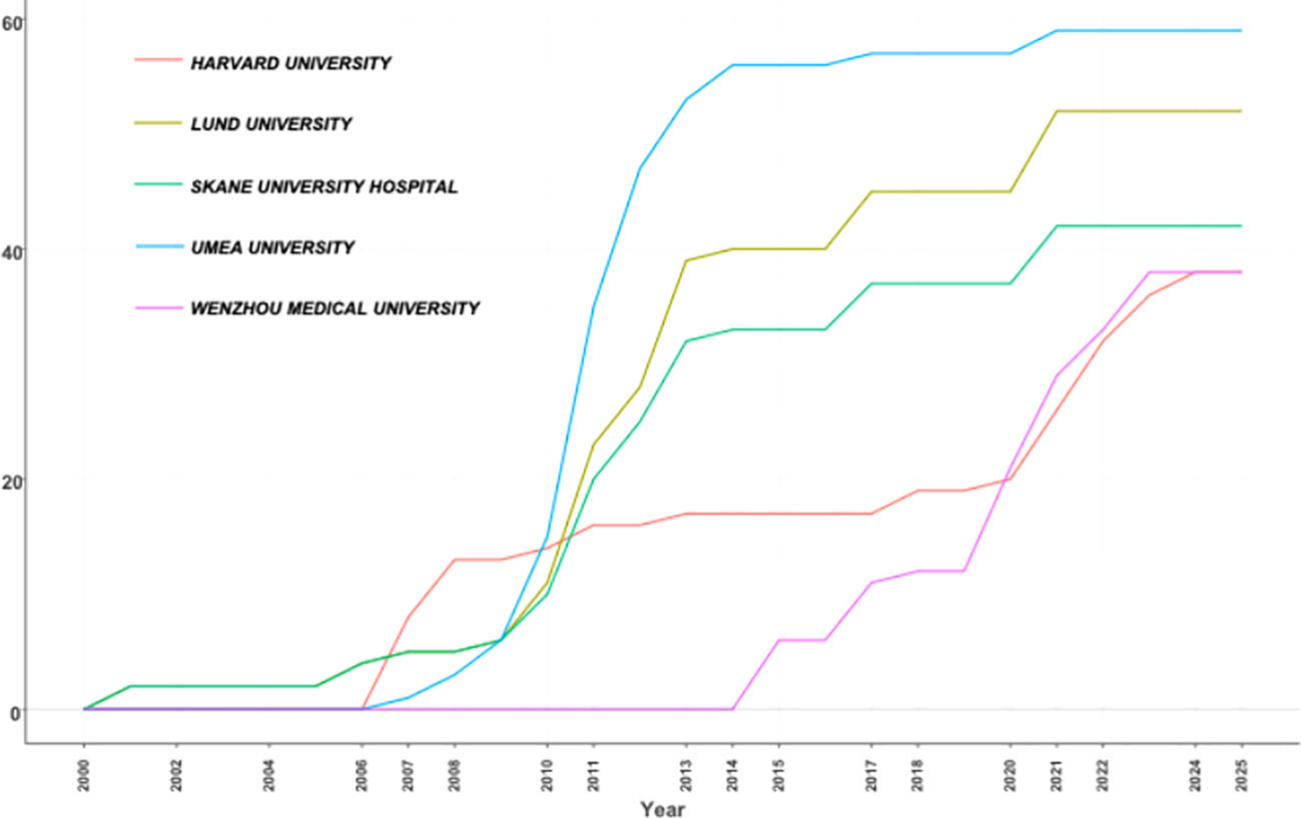
The contributions of the top 5 organizations by year.

Among a total of 1107 contributing institutions, the bibliometric analysis identified the top 20 institutions that had published at least 21 papers and quantified the publication outputs over time of the five institutions that had the highest number of publications worldwide. This analytical approach was employed to delineate the contributions of individual institutions within the field. As shown in **Table 3**, institutional origins were highly diverse, with Sweden occupying a central position in overall productivity; notably, the top three publishing institutions all originated in this country. Korea contributed the most significant number of participating institutions (N = 5), reflecting its broad engagement across the field. Umea University demonstrated the highest research activity, with 59 publications, followed by Lund University with 52. The temporal profiling presented in **Figure 4** has effectively delineated the distinct phases characterizing the trajectory of research activity in this domain. During the early foundational period spanning from 2000 to 2006, research efforts were primarily concentrated within a specific subset of institutions located in Sweden. From 2006 onward, the landscape of research participation expanded as more institutions in Sweden entered this field, and a gradual increase in participation from research entities in China and the USA further accompanied this expansion. Among these contributing regions, institutions based in Switzerland and the USA demonstrated an earlier initiation of research engagement in this area, along with a more accelerated trend of growth in their research outputs, while the contributions from Chinese institutions, in contrast, began to emerge at a relatively later stage in the overall timeline of research development.

**Table 3:** The top 20 institutions that contributed to publications in metabolic syndrome-cancer research, ranked by publication count.

Institution	Country	Counts
Umea University	Sweden	59
Lund University	Sweden	52
Skane University Hospital	Sweden	42
Harvard University	USA	38
Wenzhou Medical University	China	38
Seoul National University (SNU)	Korea	34
Tel Aviv University	Israel	31
Universita Della Campania Vanvitelli	Italy	31
University Of Oslo	Norway	30
Catholic University Of Korea	Korea	29
Sun Yat Sen University	China	26
Sungkyunkwan University (SKKU)	Korea	26
Harvard University Medical Affiliates	USA	25
Aix-Marseille Universite	France	23
Erasmus Mc	Netherlands	23
Erasmus University Rotterdam	Netherlands	23
Samsung Medical Center	Korea	22
Universidade Do Porto	Portugal	22
National Cancer Center - Korea (NCC)	Korea	21
Norwegian Institute Of Public Health (NIPH)	Norway	21

**Table 4** lists the top 10 journals, ordered by publication volume, along with their latest impact factors (IF) (29). 7 out of the top 10 journals were in the first quartile (Q1) of the Journal Citation Reports (JCR). Among these journals, the American Journal of Clinical Nutrition has received the largest number of citations, while the Journal of Clinical Oncology stands out as the one with the highest IF.

**Table 4:** The top 10 journals that contributed to publications in metabolic syndrome-cancer research, ranked by total citations.

Rank	Journal	TCs	Average TCs	Counts	IF	JCR area
1	*American Journal of Clinical Nutrition*	1214	173.43	7	6.9	Q1
2	*Cancer*	1211	86.50	14	5.1	Q1
3	*Diabetes Care*	1155	288.75	4	16.6	Q1
4	*Cancer Epidemiology Biomarkers & Prevention*	913	76.08	12	3.4	Q2
5	*Journal of Clinical Oncology*	886	295.33	3	43.4	Q1
6	*Annals of Oncology*	672	84.00	8	65.4	Q1
7	*International Journal of Cancer*	582	48.50	12	4.7	Q1
8	*Cancer Causes & Control*	510	51.00	10	2.1	Q3
9	*Obesity Research & Clinical Practice*	488	488.00	1	2.3	Q3
10	*American Journal of Epidemiology*	483	161.00	3	4.8	Q1

TC, total citation; IF, impact factor; JCR, Journal Citation Reports.

**Figure 5:**
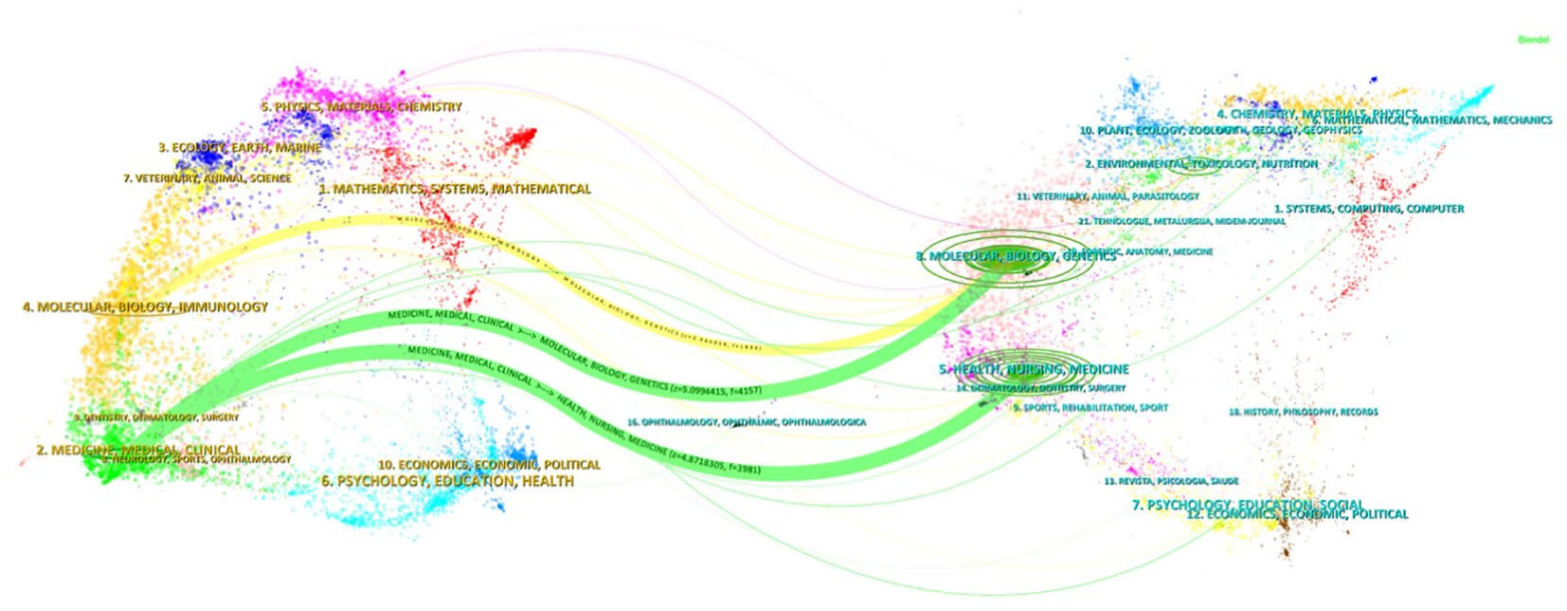
The dual-map overlays of journals.

The dual-map overlay (**Figure 5**) provides a further visualization of the disciplinary linkages between citing and cited literature, thereby revealing a diverse and intricate pattern of cross-field interactions. Specifically, research conducted within the domains of Molecular & Biology & Genetics and Health & Nursing has been extensively cited by publications belonging to the Medicine & Medical & Clinical fields, whereas the former two fields have substantially referenced literature that originates from within their own respective disciplinary boundaries.

**Figure 6:**
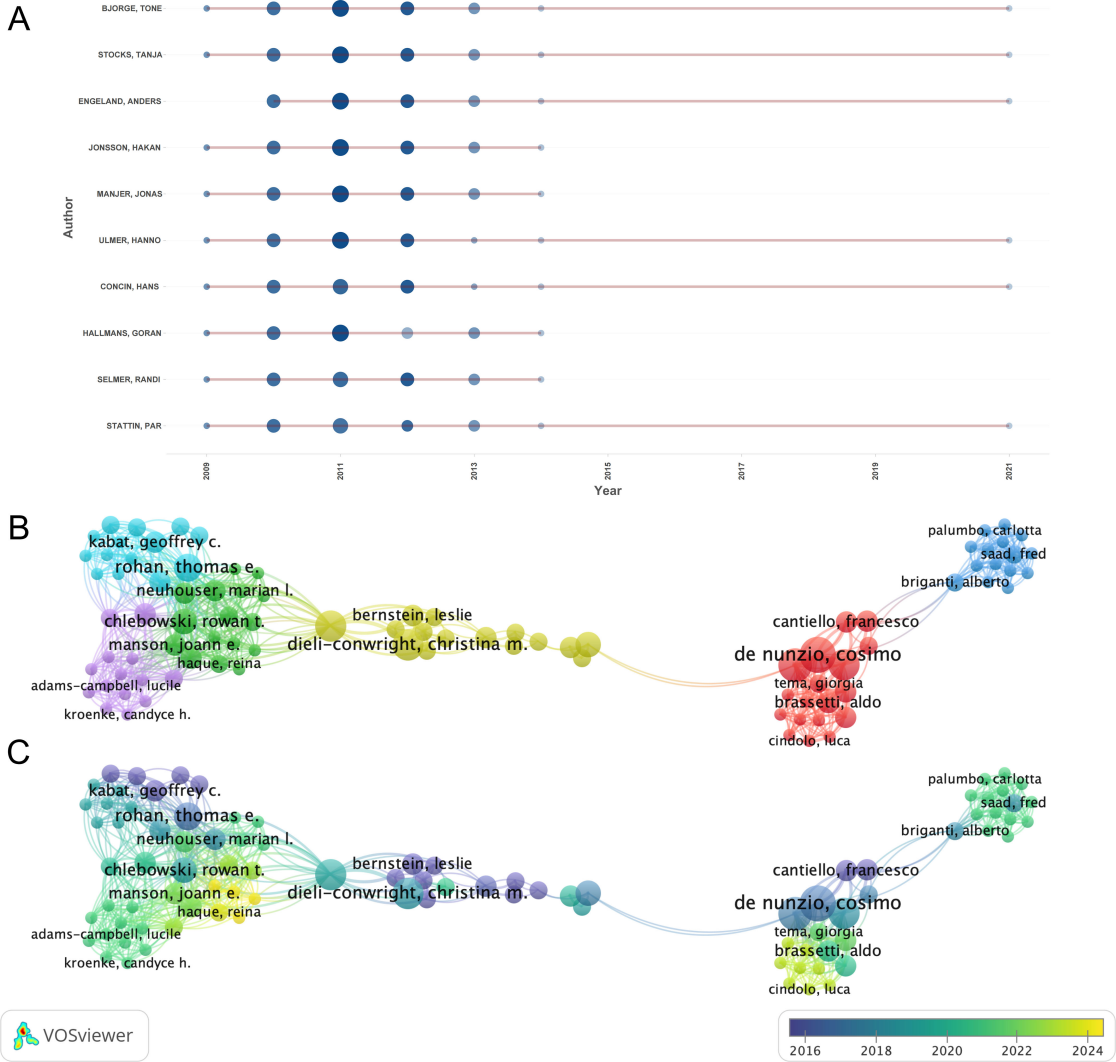
The knowledge map of active and co-cited authors, along with the situation of their article citations. **(A)** The total citations and number of articles by authors each year. **(B)** The active co-cited cluster of authors. **(C)** The active cited period of the authors. The size of each circle corresponds to the number of publications, while each color indicates a specific cluster.

The collaborative relationships among researchers are shown in **Figure 6B**. Among the total of 3804 authors, 92 authors were able to form a collaborative network and were partitioned into six clusters. These clusters segregated into two prominent communities, with relatively sparse inter-community linkages, underscoring the need for stronger collaboration among researchers studying the associations between MetS and cancer. The time-overlapping network of clustering outcomes is presented in **Figure 6C**. Annual publication and citation trends are summarized in **Figure 6A**. **Table 5** identified the ten most frequently cited authors, with Italy and Sweden each contributing three. The h-index is a quantitative metric used to evaluate academic achievements, introduced by American physicist Jorge E. Hirsch in 2005 for measuring academic impact. This metric thoroughly captures both the number and quality of research papers produced by researchers, thus reflecting their long-term contributions and influence within the area. *Tone Bjørge* and *Tanja Stocks* produced the most significant number of publications (N = 16), while *Bjørge, Stocks*, *Håkan Jönsson*, and *Jonas Manjer* shared the highest H-index (H-index = 15). Katherine Esposito and Dario Giugliano accumulated the most extensive total citations (N = 1556).

**Table 5:** The top 10 authors who contributed to publications in metabolic syndrome-cancer research, ranked by total citations.

Rank	Author	Country	TCs	H-index	Documents
1	*Katherine Esposito*	Italy	1556	8	8
2	*Dario Giugliano*	Italy	1556	8	8
3	*Paolo Chiodini*	Italy	1427	5	5
4	*Tone Bjørge*	Norway	1414	15	16
5	*Tanja Stocks*	Sweden	1414	15	16
6	*Håkan Jönsson*	Sweden	1405	15	15
7	*Jonas Manjer*	Sweden	1405	15	15
8	*Hanno Ulmer*	Austria	1374	14	15
9	*Randi M Selmer*	Norway	1317	14	14
10	*Hans Concin*	Austria	1296	13	14

### 3.2 Research hotspot analysis

#### 3.2.1 Most cited publications

Citation frequency serves as a practical indicator for identifying influential work within the field (30). **Table 6** presents the ten most frequently cited publications, each of which has accumulated more than 284 citations. Among these ten articles, three were all published before 2007. **Table 7** summarizes the most frequently co-cited references. The most influential co-cited article entitled “*Metabolic syndrome and risk of cancer: a systematic review and meta-analysis*” has provided evidence indicating that metabolic syndrome is linked to an elevated risk of developing a variety of common cancers and the degree of this increased risk differs according to factors such as sex specific characteristics of the studied population and the diagnostic criteria used to define MetS.

**Table 6:** The top 10 cited manuscripts that contributed to publications in metabolic syndrome-cancer research, ranked by total citations.

Rank	Title	Journal	Type	Publication year	TCs
1	*Metabolic syndrome and risk of cancer: a systematic review and meta-analysis*	*Diabetes Care*	Meta-Analysis	2012	973
2	*Increased oxidative stress in obesity: implications for metabolic syndrome, diabetes, hypertension, dyslipidemia, atherosclerosis, and cancer*	*Obesity Research & Clinical Practice*	Review	2013	488
3	*Sirt1 improves healthy ageing and protects from metabolic syndrome-associated cancer*	*Nature Communication*	Article	2010	481
4	*Human exposure to endocrine disrupting compounds: Their role in reproductive systems, metabolic syndrome and breast cancer. A review*	*Environmental Research*	Review	2016	457
5	*Metabolic syndrome in men with prostate cancer undergoing long-term androgen-deprivation therapy*	*Journal of Clinical Oncology*	Article	2006	456
6	*Metabolic syndrome increases the risk of primary liver cancer in the United States: a study in the SEER-Medicare database*	*Hepatology*	Article	2011	427
7	*Metabolic syndrome, hyperinsulinemia, and colon cancer: a review*	*American Journal of Clinical Nutrition*	Review	2007	416
8	*The metabolic syndrome: A high-risk state for cancer?*	*American Journal of Pathology*	Review	2006	354
9	*Tumor necrosis factor-alpha induces endothelial dysfunction in the prediabetic metabolic syndrome*	*Circulation Research*	Article	2006	294
10	*Diabetes, metabolic syndrome, and breast cancer: a review of the current evidence*	*American Journal of Clinical Nutrition*	Review	2007	284

**Table 7:** The top 10 locally cited manuscripts that contributed to publications in metabolic syndrome-cancer research, ranked by total citations.

Rank	Co-cited references	Journal	Type	Publication year	TCs
1	*Metabolic syndrome and risk of cancer: a systematic review and meta-analysis*	*Diabetes Care*	Meta-Analysis	2012	154
2	*Harmonizing the metabolic syndrome: a joint interim statement of the International Diabetes Federation Task Force on Epidemiology and Prevention; National Heart, Lung, and Blood Institute; American Heart Association; World Heart Federation; International Atherosclerosis Society; and International Association for the Study of Obesity*	*Circulation Research*	Guideline	2009	110
3	*Executive Summary of The Third Report of The National Cholesterol Education Program (NCEP) Expert Panel on Detection, Evaluation, And Treatment of High Blood Cholesterol In Adults (Adult Treatment Panel III)*	*JAMA*	Guideline	2001	102
4	*Diagnosis and management of the metabolic syndrome: an American Heart Association/National Heart, Lung, and Blood Institute Scientific Statement*	*Circulation*	Review	2005	70
5	*Metabolic syndrome and cancer risk*	*European Journal of Cancer*	Article	2008	67
6	*Prevalence of the metabolic syndrome among US adults: findings from the third National Health and Nutrition Examination Survey*	*JAMA*	Article	2002	62
7	*Metabolic syndrome and the risk of prostate cancer in Finnish men: a population-based study*	*Cancer Epidemiology, Biomarkers & Prevention*	Article	2004	62
8	*Metabolic syndrome--a new world-wide definition. A Consensus Statement from the International Diabetes Federation*	*Diabetic Medicine*	Review	2006	58
9	*Overweight, obesity, and mortality from cancer in a prospectively studied cohort of U.S. adults*	*New England Journal of Medicine*	Article	2003	56
10	The metabolic syndrome and risk of incident colorectal cancer	*Cancer*	Article	2006	55

### 3.3 Analysis of keywords

**Figure 7:**
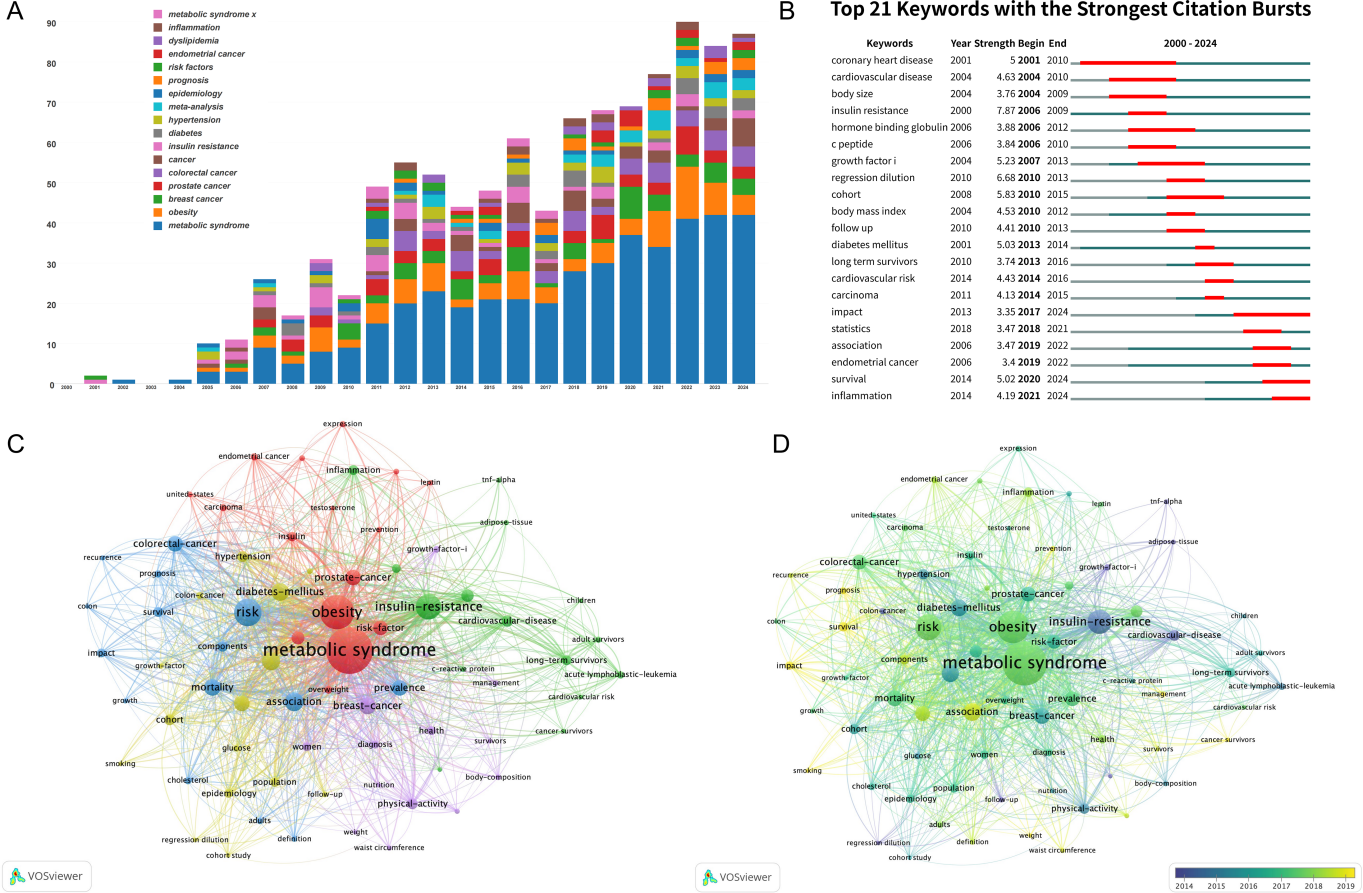
Keywords cluster map of research in the field of metabolic syndrome-cancer research. **(A)** The ratio of each keyword in the publications each year; **(B)** A compilation of the top 21 keywords with the strongest citation burst; **(C)** Clustering network of keywords co-occurrence; **(D)** Time-overlapping network of keywords co-occurrence. The size of the nodes reflects the frequency of keywords, and the distance between them signifies the strength of the relationships.

Among the total 2227 keywords, 118 keywords exceeded the threshold of 10 occurrences following the merge of synonymous terms. **Figure 7A** presents the top 17 keywords sorted by their frequency of occurrence, among which “metabolic syndrome” ranks as the most frequently used keyword, followed by “obesity”. **Figure 7B** illustrates that 21 keywords exhibit citation bursts with a duration of at least one year. Among these keywords, “Coronary heart disease” shows not only the strongest citation burst but also the longest-lasting one. In contrast, terms like “impact” and “survival” display citation bursts that are comparably strong yet emerged more recently. It is worth noting that “insulin resistance” stands out as the most prominent keyword, with an early citation burst that reached its peak between 2006 and 2009. Closely associated keywords were aggregated into thematic clusters, as shown in **Figure 7C**, which resolved five distinct clusters. Cluster 1, marked in red, predominantly centers on metabolic dysfunction as a key driver of hormone-related cancers. Cluster 2, distinguished by green, places emphasis on cardiometabolic inflammation and the long-term survivorship of individuals affected by relevant conditions. Cluster 3, represented by purple, focuses on lifestyle-modifiable factors that contribute to cancer risk. Cluster 4, indicated in yellow, reflects epidemiological evidence that establishes links between core metabolic traits and the development of cancer within large cohort research frameworks. Cluster 5, identified by blue, characterizes outcome-oriented research that evaluates mortality, survival rates, and the quantification of risk within the metabolic-cancer axis. **Figure 7D** illustrates the temporal overlap exhibited by these keywords. Research conducted in the early stage, represented by the color blue, focused primarily on specific metabolic abnormalities and types of cancer, with examples including “diabetes mellitus”, “hypertension”, “insulin resistance”, and “breast cancer”. During the mid-stage of development, denoted by green, the research focus shifted toward conceptual entities, with “metabolic syndrome” being a typical example of such entities. In contrast, recent research work, indicated by yellow, has placed increasing emphasis on topics related to treatment and prognosis, with examples including “impact”, “survival”, and “prevention”.

**Figure 8:**
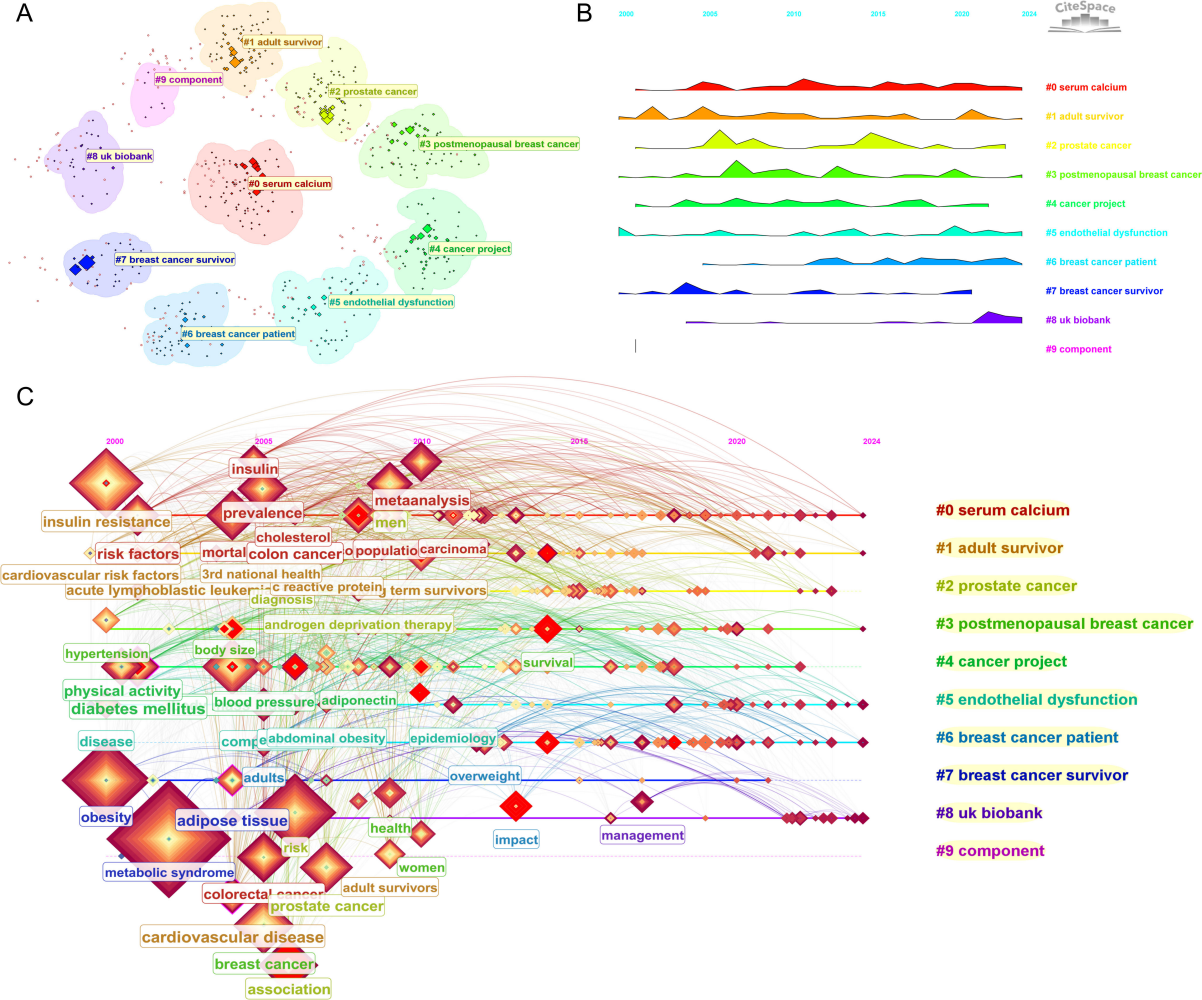
The distribution of the keywords by year. **(A)** The overlapping map of the lusters of 15 keywords illustrates their co-occurrence. **(B)** The map of the frequency of the appearance of the 15 keywords over time. **(C)** The map displays the frequency of appearance of all keywords by year.

**Figure 8** provides a more precise visualization of these bursts. **Figure 8A** illustrates the clusters formed by co-occurring keywords. In **Figure 8B**, apart from cancer-related topics that have shown intermittent bursts from 2000 to 2024, the keyword “UK Biobank” appeared in 2021 and quickly intensified to develop into a leading research focus. **Figure 8C** illustrates that the activity reached its peak between 2000 and 2010, during which time studies focused on the core metabolic features underlying MetS. Among them, Postmenopausal, a keyword related to age, is also worthy of attention. With the aging of the global population, relevant literature shows a development trend.

**Figure 9:**
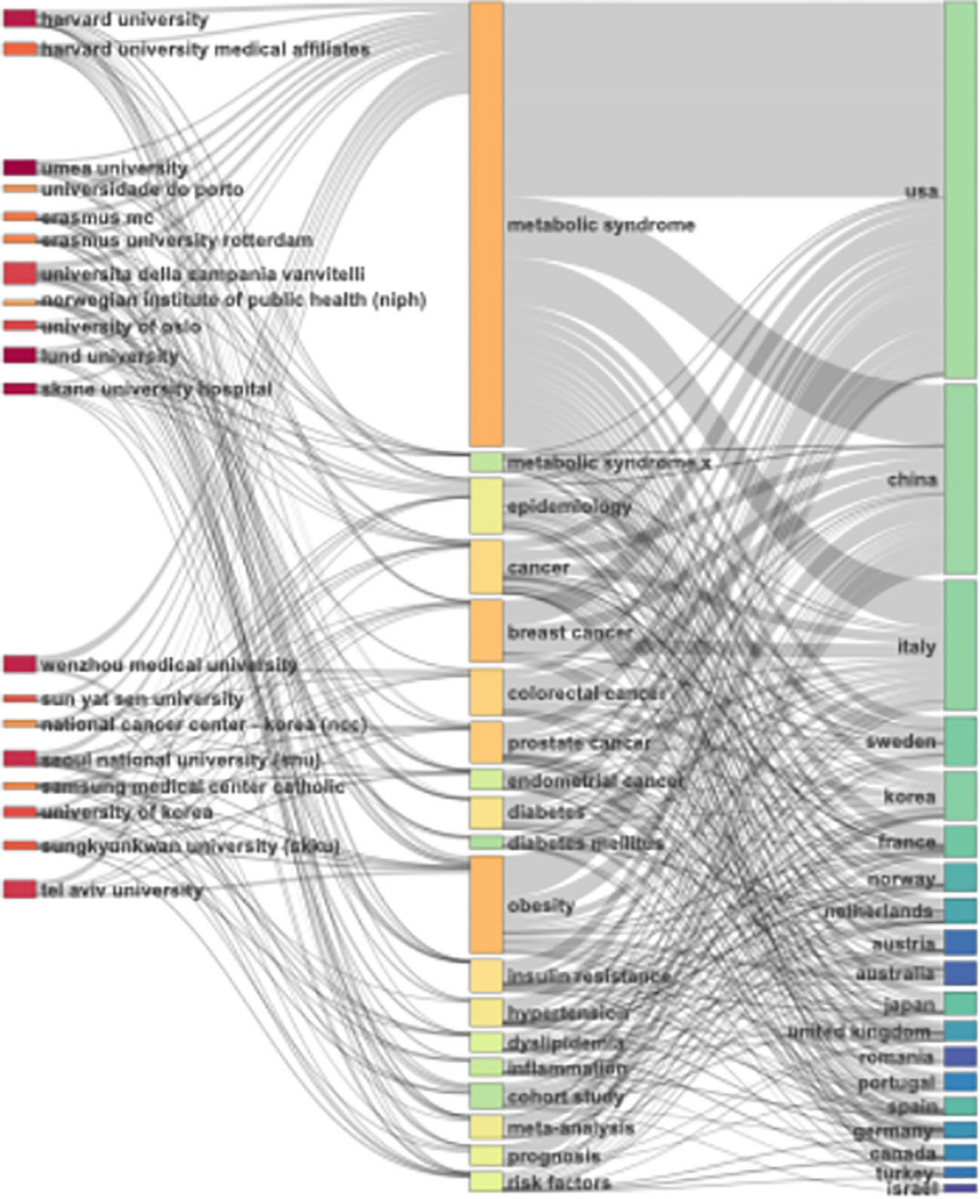
Network visualization between the top countries, organizations, and keywords. The thickness of the lines signifies the relative frequency of co-occurrence within a certain timeframe.

The Sankey diagram (**Figure 9**) visualizes the flow of research themes across different institutions and countries, thereby mapping the transnational redistribution of research focus within the research landscape encompassing MetS and cancer. It is reasonable to hypothesize that while institutions in Europe and North America have placed mainly emphasis on mechanistic investigations, institutions in China have predominantly advanced research in the fields of epidemiology and phenotypic studies related to cancer and its associated metabolic comorbidities.

### 3.4 Clinical trials landscapes

Research retrieved from PubMed extends the findings from a clinical perspective, complementing the results obtained through Web of Science (WoS). **Table 8** summarizes seventeen clinical trials indexed in PubMed, collectively delineating the influence of MetS on cancer susceptibility, therapeutic responsiveness, and patient outcomes across the entire continuum of oncogenesis, treatment, and survivorship. These investigations span a broad spectrum of malignancies, with breast cancer being the most frequently represented entity (n = 6); leukemia, biliary tract cancer, colorectal carcinoma, gastric cancer, and prostate cancer are also included.

**Table 8:** Clinical trials of MetS and cancer indexed in PubMed.

Number	Cancer type	Title	Research problem	Results	Research design classification	Clinical trial phases	Journal	PMID
1	*Leukemia*	*Obesity and metabolic syndrome in adolescent survivors of standard risk childhood acute lymphoblastic leukemia in Saudi Arabia.*	Relationship of unhealthy weight status in adolescent ALL survivors and MetS.	Half of the sample had unhealthy weight status. The prevalence of MetS was 7.1% among individuals aged 9 years and older. However, MetS was present in 19% of the overweight and obese survivors, and 7.1% of the sample had at least two of the components of MetS.	Cross-sectional study	N/A	*Pediatric blood & cancer*	22162511
2		*Acute Activation of Metabolic Syndrome Components in Pediatric Acute Lymphoblastic Leukemia Patients Treated with Dexamethasone.*	Relationship of dexamethasone treatment and activation of MetS components.	Dexamethasone treatment significantly elevated the following fasting serum levels, increased insulin resistance from 8% to 85%, and raised both diastolic and systolic blood pressure.	Prospective observational study	N/A	*PloS One*	27362350
3		*Prevalence and characteristics of metabolic syndrome in adults from the French childhood leukemia survivors’ cohort: a comparison with controls from the French population.*	Relationship of childhood leukemia treatment and MetS risk.	MetS was observed in 10.3% of patients and 4.5% of controls. Patients who received total body irradiation transplant had the highest risk; other treatment groups also showed a greater risk than controls, including patients treated only with chemotherapy. Odds Ratios were 1.68 for chemotherapy alone, 2.32 for chemotherapy combined with cranial irradiation, and 2.18 for patients transplanted without irradiation. Total body irradiation recipients with MetS displayed a distinct profile compared to controls, marked by smaller waist circumference and higher levels of triglycerides, fasting glucose, and systolic blood pressure. Conversely, recipients of cranial irradiation with MetS had a larger waist circumference than the controls. Regardless of anti-leukemic treatment, the risk of MetS was higher among childhood leukemia survivors.	Prospective cohort study	N/A	*Haematologica*	29351982
4	*Biliary Tract Cancer*	*The metabolic syndrome and risk factors for biliary tract cancer: a case-control study in China*	Relationship of MetS and biliary tract cancer risk.	HBsAg+/anti-HBc+ individuals with a history of diabetes, cholelithiasis, TC, and HDL were significantly related to ICC. Cholelithiasis, Tri, LDL, diabetes, Apo A, and Apo B were significantly associated with ECC. Diabetes, cholelithiasis, and Apo A were strongly inversely correlated with GC.	Case-control study	N/A	*Asian Pacific Journal of Cancer Prevention*	22901155
5	*Breast Cancer*	*Effect of combination exercise training on metabolic syndrome parameters in postmenopausal women with breast cancer.*	Relationship of combination exercise training and MetS parameters in postmenopausal women with breast cancer.	Significant differences were observed for VO2peak, RHR, BW, BMI, WHR, SBP, fasting insulin and glucose, HDL-C and TG between experimental and control groups after 15 weeks (P< 0.05).	RCT protocol	N/A	*Journal of Cancer Research and Therapeutics*	22842368
6		*Effects of Aerobic and Resistance Exercise on Metabolic Syndrome, Sarcopenic Obesity, and Circulating Biomarkers in Overweight or Obese Survivors of Breast Cancer: A Randomized Controlled Trial*	Relationship of a 16-week supervised progressive combined exercise intervention and components of MetS.	The postintervention MetS z-score was significantly improved in the exercise group compared to the usual care group. Sarcopenic obesity and circulating biomarkers, including insulin, IGF-1, leptin, and adiponectin, showed significant improvement postintervention compared with usual care. At the 3-month follow-up, all MetS variables remained significantly improved compared with baseline in the exercise group.	RCT(behavioral intervention)	N/A	*Journal of Clinical Oncology*	29356607
7		*Metabolic syndrome and breast cancer risk: a case-cohort study nested in a multicentre Italian cohort.*	Relationship of MetS to breast cancer risk	The presence of MetS was associated with significantly increased breast cancer risk in all women. When the analyses were repeated separately for menopausal status, the association was limited to postmenopausal women and absent in premenopausal women. Of MetS components, only high blood glucose was significantly associated with increased breast cancer risk in all women and postmenopausal women, but not premenopausal women.	Nested case-cohort study	N/A	*PloS One*	26030767
8		*Effect of exercise on metabolic syndrome in black women by family history and predicted risk of breast cancer: The FIERCE Study.*	Relationship of aerobic exercise and MetS in women at high risk of breast cancer.	Among women with a family history of breast cancer, the exercise arms had lower mean MetS z scores, which suggested an improvement in the metabolic profile, than controls at 6 months (controls, + 0.55; home-based arm, -0.97, P <.01; supervised arm, -0.89, P <.01). Stratified analyses by projected breast cancer risk suggested similar but statistically nonsignificant findings, with those at high risk having more favorable changes in the MetS z score in the exercise arms versus the control arm. These changes were primarily attributed to changes in blood pressure, triglycerides, and HDL levels.	RCT	Phase II	*Cancer*	29975403
9		*Low-fat dietary pattern and breast cancer mortality by metabolic syndrome components: a secondary analysis of the Women’s Health Initiative (WHI) randomised trial.*	Relationship of low-fat dietary pattern and breast cancer mortality in women with MetS components.	HRs and 95% confidence intervals (CI) for dietary intervention influence on death from breast cancer were with no MS components (n = 10,639), HR 1.09, 95% CI 0.63-1.87; with 1–2 MS components (n = 30,948), HR 0.80, 95% CI 0.62-1.02; with 3–4 MS components (n = 4,246), HR 0.31, 95% CI 0.14-0.69 (interaction P = 0.01).	Secondary analysis of RCT	Phase III	*British Journal of Cancer*	34006923
10		*Breast cancer incidence and mortality by metabolic syndrome and obesity: The Women’s Health Initiative.*	Relationship of MetS and breast cancer incidence and mortality	After a >20-year mortality follow-up, a higher MetS score, adjusted for BMI, was significantly associated with poorer prognosis, ER-positive, PR-negative cancers, 53% more deaths after breast cancer, and 44% higher breast cancer mortality. Obesity status, adjusted for MetS score, was significantly associated with better prognosis, ER-positive, PR-positive cancers, more total breast cancers, and more deaths after breast cancer, with higher breast cancer mortality only in women with severe obesity.	Secondary analysis of prospective cohort	Phase III	*Cancer*	38736319
11	*Colorectal Cancer*	*Components of metabolic syndrome and metachronous colorectal neoplasia.*	Relationship between MetS components and risk of metachronous colorectal neoplasia.	MetS classification was associated with increased odds of metachronous neoplasia among women but not among men. High waist circumference in men and women, as well as elevated fasting glucose in women, were associated with increased odds, whereas none of the other criteria were independently related to metachronous neoplasia. Exploratory analysis of waist circumference and fasting glucose suggested an interaction, where only the combination of large waist circumference and elevated glucose conferred significantly increased odds of metachronous neoplasia among both men.	Prospective cohort study	N/A	*Cancer epidemiology, biomarkers & prevention*	19318435
12	*Gastric Cancer*	*A randomized phase II trial of preoperative exercise to reduce operative risk in gastric cancer patients with metabolic syndrome: adjuvant exercise for general elective surgery (AEGES) study group*	Relationship of preoperative exercise training and perioperative complication risk in gastric cancer patients with MetS.	The anticipated primary result is that the preoperative exercise group is expected to reduce the incidence of perioperative complications from a historical rate of 35% (in obese patients) to 20%. Key secondary outcomes anticipated to improve include reduction in body weight, increase in HDL cholesterol, shorter operation time, decreased intraoperative blood loss, and an adequate number of dissected lymph nodes.	RCT protocol	Phase II	*Japanese journal of clinical oncology*	18202030
13		*Matched pair analysis to examine the effects of a planned preoperative exercise program in early gastric cancer patients with metabolic syndrome to reduce operative risk: the Adjuvant Exercise for General Elective Surgery (AEGES) study group.*	Relationship of planned preoperative exercise program to postoperative complications of gastric cancer	Data from a total of 72 patients (54 in the surgery-alone group, 18 in the preoperative exercise group) were analyzed. The median operative time and amount of bleeding were 208 min and 130 ml in the surgery-alone group and 248 min and 105 ml in the exercise group, respectively. Postoperative complications occurred in one case (5.5%) in the exercise group and 22 (40.7%) cases in the surgery-alone group.	RCT	Phase II	*Annals of surgical oncology*	24671637
14	*Prostate Cancer*	*Metabolic syndrome-like components and prostate cancer risk: results from the Reduction by Dutasteride of Prostate Cancer Events (REDUCE) study.*	Relationship of MetS-like components and prostate cancer risk	In all, 2171 men (34%) had one MetS-like component, 724 (11%) had two, and 163 (3%) had three or four. Men with more MetS-like components had lower PSA levels. One vs no MetS-like components was protective for overall prostate cancer and low-grade prostate cancer. Two or three to four MetS-like components were not significantly related to prostate cancer. While one MetS-like component was unrelated to high-grade prostate cancer, two or three to four MetS-like components were associated with increased high-grade prostate cancer risk, although only the latter was significant.	Secondary analysis of RCT	Phase III	*BJU International*	24931061
15		*Impact of resistance training on body composition and metabolic syndrome variables during androgen deprivation therapy for prostate cancer: a pilot randomized controlled trial.*	Relationship of resistance training and body composition/MetS variables during androgen deprivation therapy for prostate cancer.	Post-intervention, EXE significantly improved lean mass, sarcopenia prevalence, body fat %, strength, and prostate cancer-specific quality of life compared to NoEXE. No significant differences were observed between groups for physical function or MetS-related variables except waist circumference.	Pilot RCT	N/A	*BMC cancer*	29614993
16		*Metabolic Syndrome is Associated with Prostate Cancer Diagnosed on Biopsy but not the Gleason Score and the Number of Cancer-Positive Cores: A Prospective Controlled Study.*	Relationship of MetS and prostate cancer in patients undergoing prostate biopsy	A total of 908 men underwent biopsies, of which 492 (51.5%) had MetS. The number of patients diagnosed with PCa in biopsy was 270 (29.7%). PCa cases were significantly older, with a lower prostate volume and a higher PSA value and higher blood pressure compared to patients without PCa. 146 of 416 (35.0%) patients with MetS had PCa, while 124 of 492 (25.2%) patients without MetS had PCa. Out of 270 patients with PCa, 174 (64.4%) had a Gleason score <7, and 96 (35.6%) had a Gleason score ≥7. In patients with a Gleason score ≥7, PSA, DRE (+), and the number of core-positive biopsies were significantly higher compared to patients with a Gleason score <7, while glycemia and HDL cholesterol levels were significantly lower. Multivariate analysis showed that age, PSA, positive DRE, prostate volume, diastolic blood pressure, CRP, and MetS were the only independent parameters associated with a higher risk of cancer on biopsy.	Prospective controlled study	N/A	*Archivos españoles de urología*	37867335
17		*Effectiveness of a Nurse-Led Mobile-Based Health Coaching Program for Patients With Prostate Cancer at High Risk of Metabolic Syndrome: Randomized Waitlist Controlled Trial.*	Relationship of nurse-led education and MetS risk in patients with PC undergoing ADT.	The experimental group exhibited more positive changes in the healthy lifestyle score, the level of each MetS component, body composition, and the urinary irritative and obstructive domains of health-related quality of life over time than the waitlist control group.	RCT(behavioral intervention)	N/A	*JMIR mHealth and uHealth*	38300697

N/A, not applicable. Phase classification was applied only to interventional randomized controlled trials when explicitly reported. Observational studies (including cross-sectional, cohort, nested case-cohort, and case-control studies) and behavioral/lifestyle intervention studies are not conventionally assigned clinical trial phases.

Accumulating evidence suggests that hyperglycemia, visceral adiposity, and insulin resistance, which are the cardinal disturbances of MetS, create a chronic inflammatory and hormone-altered milieu that often predisposes individuals to heightened cancer risk, suboptimal treatment tolerance, and unfavorable prognosis. In this context, metabolic-modulating pharmacotherapies have been shown to mitigate cancer risk and improve outcomes by targeting these fundamental pathological processes. Moreover, combined aerobic and resistance exercise regimens, together with low-fat, health-oriented dietary patterns, effectively ameliorate MetS parameters, ultimately reducing risk, limiting complications, and enhancing survival.

### 3.5 Key mechanisms linking MetS to cancer

**Figure 10:**
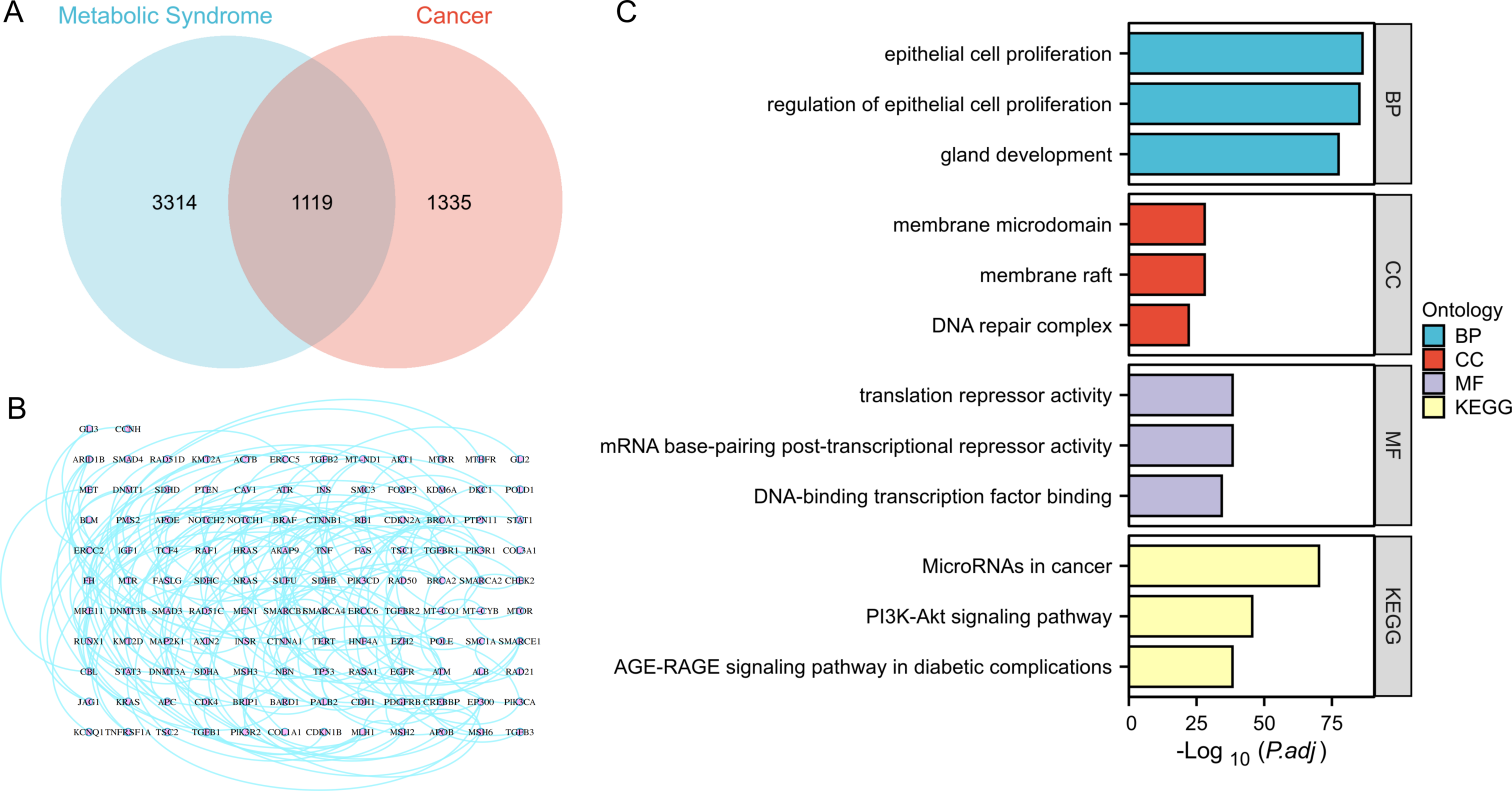
The results of enrichment analysis. **(A)** Common genes of metabolic syndrome and cancer. **(B)** Network map of PPI interaction network analysis through the STRING database. **(C)** Functional enrichment analysis of metabolic syndrome-cancer research. PPI, Protein-Protein Interaction.

After screening genes with a relevance score threshold> 10, the integrated analysis of 4433 MetS-related genes and 2454 cancer-associated genes revealed 1119 overlapping genes (**Figure 10A**). Detailed gene information for MetS and cancer can be found separately in **Supplementary Tables 1**, **2**. The overlapping gene list is presented in **Supplementary Table 3**. To delineate the molecular architecture underlying the interaction landscape between MetS and cancer, a protein–protein interaction (PPI) analysis was performed. A total of 1,119 shared targets were first uploaded to the STRING (Search Tool for the Retrieval of Interacting Genes/Proteins) database (detailed results are presented in **Supplementary Table 4**), from which a high-confidence PPI network (confidence score ≥ 0.90) was generated. The resulting network consisted of 122 nodes interconnected by numerous edges, reflecting the intricate interplay among these putative targets (**Figure 10B**).

The GO enrichment results show significant BP enrichment in the regulation of epithelial cell proliferation and gland development. CC enrichment was found in membrane microdomains and the DNA repair complex, suggesting localization in these structures. MF enrichment included transcriptional repressor activity and DNA-binding transcription factor binding. The KEGG pathway analysis revealed significant enrichment of the PI3K-Akt and AGE-RAGE signaling pathways in diabetic complications (**Figure 8C**; detailed results are provided in **Supplementary Table 5**).

**Figure 11:**
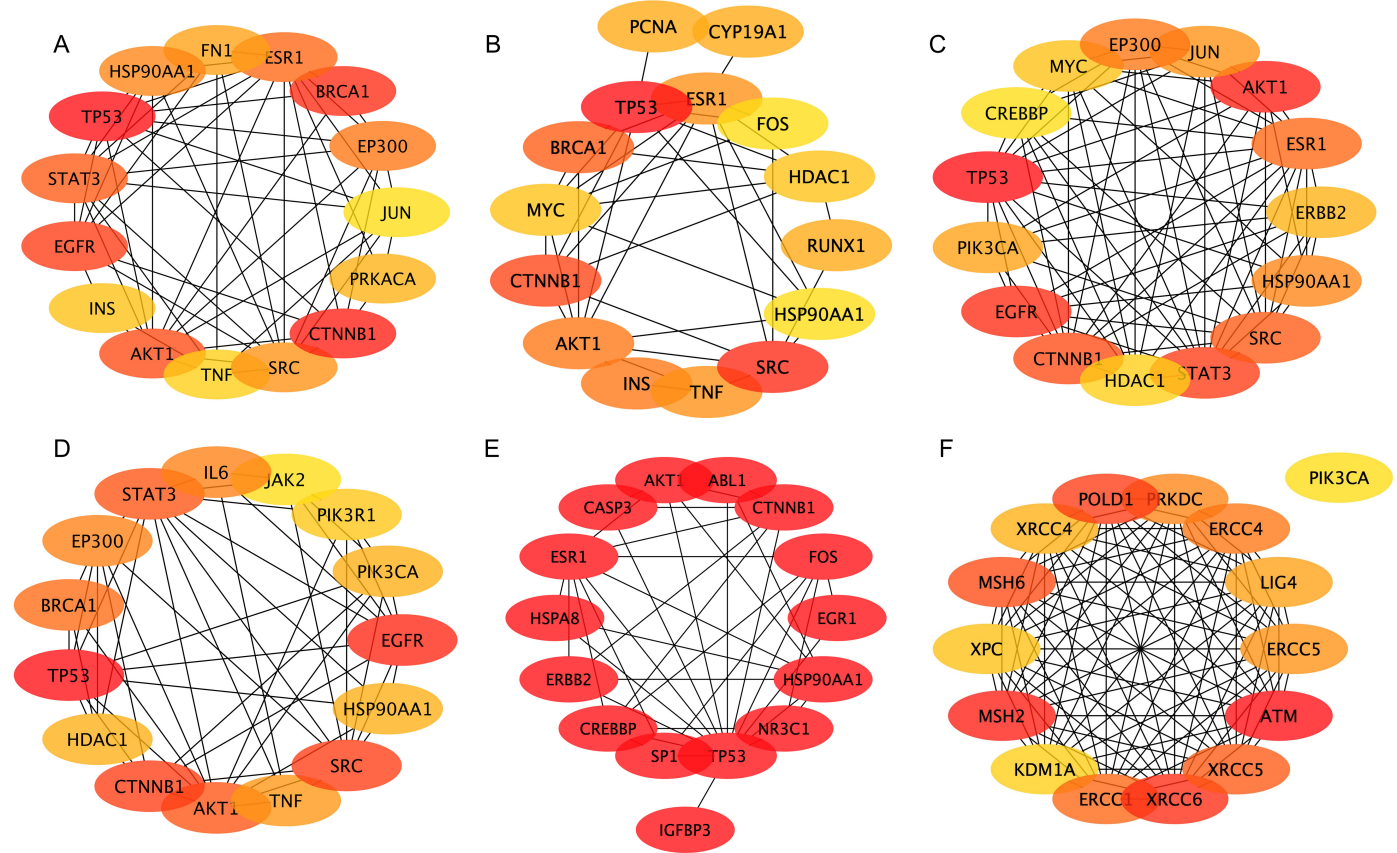
The results of hub genes were calculated using the Cytoscape plugin CytoHubba with 6 different algorithms. **(A)** Betweenness **(B)**, BottleNeck **(C)**, Closeness **(D)**, Degree **(E)**, Eccentricity **(F)**, MCC (Maximum Clique Centrality).

To pinpoint the principal nodes embedded within the PPI network, based on the 1119 shared genes, an in-depth evaluation was conducted using the cytoHubba module in Cytoscape. A positive correlation exists between the proximity of a color to red and the degree of a hub gene, such that colors closer to red correspond to a higher degree of the hub gene. By systematically examining topological metrics across six complementary algorithms (Betweenness, BottleNeck, Closeness, Degree, EcCentricity, and MCC), TP53, BRCA1, CTNNB1, SRC, AKT1, EGFR, STAT3, and ATM were robustly identified as central hub targets within the PPI network (**Figure 11**). These hub genes may play key roles in the shared pathophysiological mechanisms of MetS and cancer, offering potential targets for further functional research and therapeutic development.

## 4 Discussion

### 4.1 Bibliometric characteristics

This study employed bibliometric approaches to quantify the research activity in and delineate the scholarly landscape of studies investigating the relationship between MetS and cancer over the period from 2000 to 2024.

Early epidemiological work in the late 1990s and early 2000s first signaled that core components of MetS, particularly obesity and diabetes, were associated with higher cancer incidence. As evidence accumulated, interest shifted from discrete metabolic abnormalities to viewing MetS as an integrated clinical construct that may amplify oncogenic risk. Seminal contributions by Eugenia and Rudolf (2004) consolidated cohort and case–control evidence, showing strong associations between excess adiposity and increased morbidity and mortality across colorectal, renal, endometrial, postmenopausal breast, and esophageal adenocarcinomas (31). These findings helped frame MetS as a unified pathological entity rather than a collection of isolated risk factors. Between 2006 and 2012, the field expanded rapidly, supported by large cohort studies and meta-analyses. Convergent data reinforced that MetS represents an independent risk factor for multiple solid tumors, with broad reproducibility across study populations.

A landmark systematic review and meta-analysis published in *Diabetes Care* (2012) by Esposito and colleagues synthesized 43 studies and reported robust associations between MetS and several malignancies (32). Data reflect that *Diabetes Care* ranks third among contributing journals, and three authors from this study are among the top 10 most productive researchers in the field. MetS was linked to increased risks of hepatocellular, colorectal, and bladder cancers in men, and to endometrial, pancreatic, postmenopausal breast, and colorectal cancers in women. These sex-specific patterns suggest that MetS may exert synergistic effects, particularly within hormone-responsive tumors.

Since 2013, the research landscape has broadened through the use of large datasets, more refined analytical strategies, and subgroup analyses by sex, ethnicity, and geographic setting. A 2020 meta-analysis by *Xin-Yuan Sun* et al. reported a 49% increase in breast cancer risk associated with MetS, with higher risk estimates in postmenopausal women carrying multiple MetS components (33). Similarly, *Hu* et al. (2021) identified a dose–response relationship between the number of MetS components and colorectal adenoma risk (34). The upward trend in the number of relevant publications reached its peak in 2022, a phenomenon that the COVID-19 pandemic may have shaped. During this global health crisis, the parallels between the chronic low-grade inflammation associated with MetS and the dysregulated cytokine responses triggered by SARS-CoV-2 garnered increased scholarly attention. Considering the well-established connection between cancer development and persistent inflammatory processes, this specific period further heightened research interest in the intricate interplay of the metabolic–inflammatory–immune axis, which emerged as a critical pathway linking multiple disease states of high clinical relevance.

Geographically, the top 12 contributing countries accounted for 76.18% of all entries, with 515 publications. China and the USA led in output, whereas Sweden appeared as the most interconnected collaborator. Italy and Sweden each contributed three of the top 10 most frequently cited authors, underscoring the field’s global breadth. The pattern suggests that sustained international collaboration remains critical for advancing scientific quality.

Institutional contributions provide additional insight. Sweden, characterized by strong international partnerships and high citation rates, is home to three of the top-ranking institutions. South Korea, although ranked third in national output, has the largest number of institutions (n=5) among the top 20. By contrast, despite China and the USA producing high overall volumes, their publications are dispersed across many institutions, leading to fewer entries in the top 20. This distribution indicates that concentrated, strategically networked research environments may be more influential than sheer publication volume.

Journal analysis highlights publication trends and potential dissemination venues. The *American Journal of Clinical Nutrition* has the highest citation count, while *Annals of Oncology* possesses the highest impact factor (IF = 65.4). Although China contributes substantially to publication volume, the top 10 journals are predominantly based in the USA and Europe, revealing a gap in high-impact Asian publishing platforms. Strengthening internationally visible journals in Asia may help elevate the regional influence of research.

Citation metrics offer a complementary measure of impact. The top 10 most cited articles primarily consist of systematic reviews that define key thematic directions or introduce new perspectives. Notably, average citation counts do not correspond strictly to journal impact factors, suggesting that research prominence depends on more nuanced statistical patterns rather than journal metrics alone.

Keyword co-occurrence analysis identifies “Metabolic Syndrome” and “Obesity” as the most frequent terms, while “UK Biobank” shows a rapid rise in usage. As a large prospective cohort with deep phenotyping and multi-omics datasets, the UK Biobank has enabled analyses of MetS risk in relatively rare cancers, such as biliary and pancreatic malignancies, allowing for a more precise evaluation of the causal mechanisms linking MetS components to cancer development. Effective use of such resources is expected to shift the field from descriptive associations toward mechanistic insight, improving research efficiency and supporting long-term prognostic modeling.

### 4.2 Research hot spots and frontiers

**Figure 12:**
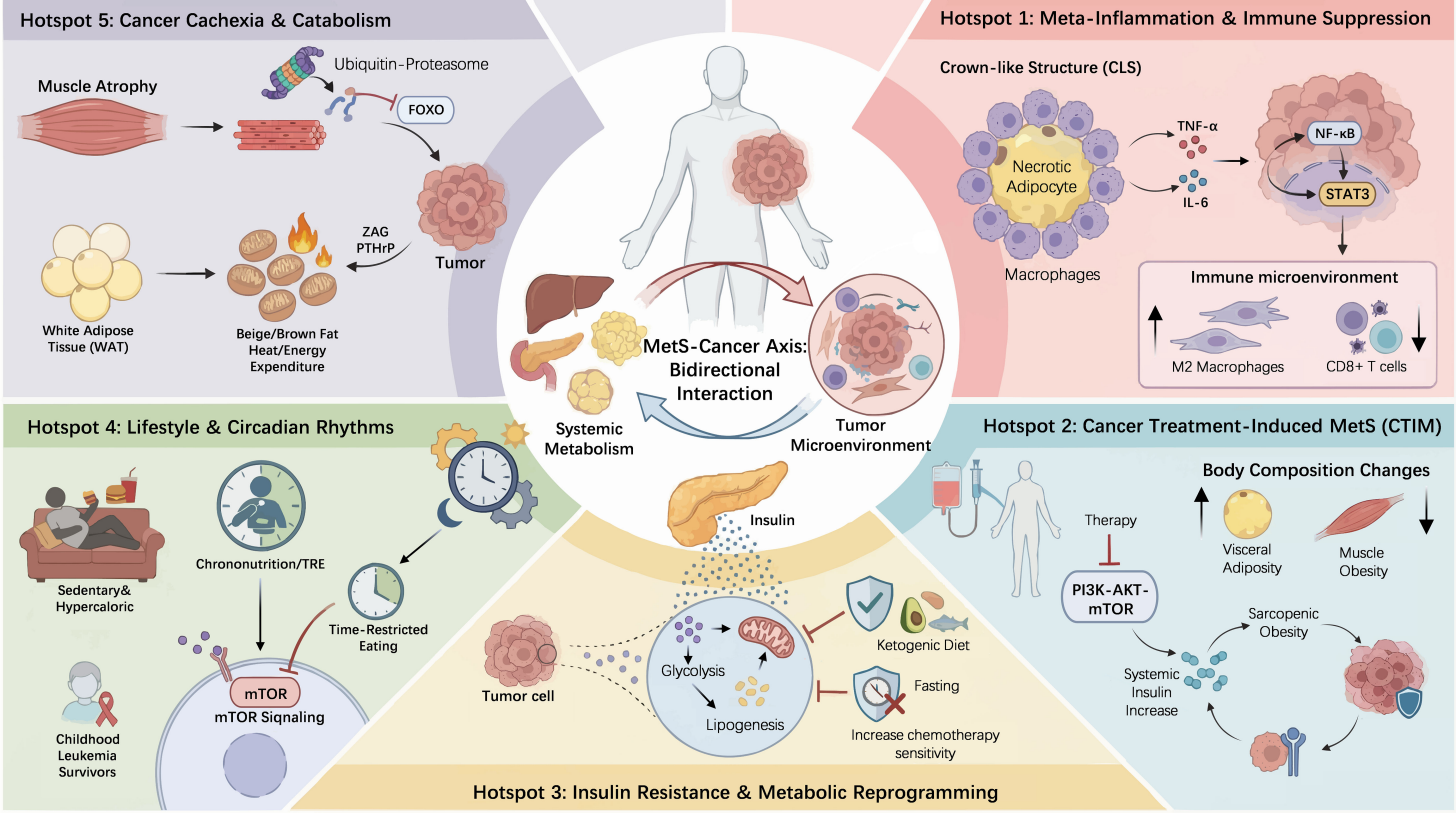
Research hotspots and frontiers.

The bibliometric hotspots identified in this study were broadly consistent with the hub genes highlighted by the bioinformatic analysis (**Figure 12**). In particular, the inflammation-related clusters corresponded closely to STAT3 and EGFR, which are central mediators of cytokine and growth factor signaling, whereas the hotspot of insulin resistance and dysregulated glucose metabolism was closely linked to AKT1-centered PI3K/AKT signaling. TP53 also connects these themes by regulating metabolic stress responses, cell survival, and tumor progression. This convergence suggests that the major research hotspots and the PPI-derived core targets reflect a shared inflammatory-metabolic network underlying the MetS-cancer association.

#### 4.2.1 Hotspot 1: Inflammation as the principal biological axis linking MetS and cancer

Chronic low-grade inflammation, often referred to as meta-inflammation, serves as the core biological axis that establishes a functional link between MetS and the process of carcinogenesis. Foundational studies have established that inflammatory signaling is a key driver of the tumor-promoting microenvironment (35). This systemic inflammatory state in MetS patients disrupts the homeostatic balance of cytokines, fostering a permissive niche for tumor initiation (36). This chronic low-grade inflammation is also conceptualized as “Inflammaging”, where age-related immune aging cannot eliminate metabolic stress, thus forming a continuous cancer-promoting environment.

Obesity-induced adipose tissue remodeling acts as a pivotal mechanism. The formation of crown-like structures (CLS), in which macrophages encircle necrotic adipocytes, generates a persistent inflammatory milieu characterized by elevated TNF-α and IL-6 (16). These mediators, alongside dysregulated adipokines, activate oncogenic transcription factors such as NF-κB and STAT3, thereby promoting cell proliferation and survival (16, 37). This was also supported by the hub-gene analysis, in which STAT3 emerged as a key inflammatory effector, while EGFR and AKT1 may act as important downstream signaling nodes linking inflammatory stimulation to tumor cell proliferation, survival, and metabolic adaptation. Recent research has further elucidated how this inflammatory cascade facilitates metastasis via immune suppression. In the context of colorectal cancer liver metastasis, the metabolic reprogramming driven by MetS reshapes the stromal architecture and immune landscape. The study demonstrated that a lipid-rich environment promotes the polarization of macrophages toward the tumor-promoting M2 phenotype while impairing CD8+ T cell surveillance (15). Recent advances in multi−omics approaches and artificial intelligence have enabled the integration of transcriptomic, metabolomic and immune profiling to delineate how inflammatory signals intersect with metabolic reprogramming and core gene networks in cancer progression (38). This convergence of metabolic dysregulation and immune evasion creates conditions that facilitate the metastasis of cancerous lesions, explaining the poor prognosis observed in metabolically dysregulated hosts.

#### 4.2.2 Hotspot 2: cancer treatment-induced MetS

While MetS drives tumorigenesis, a reciprocal relationship exists in which oncological therapies can precipitate iatrogenic metabolic dysfunction, Cancer Treatment-Induced Metabolic Syndrome (CTIM). Early mechanistic analyses revealed that cytotoxic agents, targeted therapies, and hormonal treatments can collectively disrupt glucose homeostasis, accelerate visceral adiposity, and impair insulin signaling, thereby inducing a metabolic phenotype that closely mirrors classical MetS (39). Ariaans et al. also posit that treatment-emergent insulin resistance is a direct pharmacologic consequence. Agents targeting the PI3K-AKT-mTOR pathway, in combination with glucocorticoids and platinum compounds, profoundly disrupt glucose homeostasis and mitochondrial function (39). Chemotherapy and endocrine therapy significantly alter body composition, promoting visceral adiposity while depleting muscle mass, which causes sarcopenic obesity. This shift accelerates the onset of hypertension, dyslipidemia, and hyperglycemia (17). Foundational research by Keating et al. corroborated this in prostate cancer, demonstrating that androgen deprivation therapy (ADT) significantly elevates the risk of incident diabetes and cardiovascular disease (40). This metabolic toxicity creates a feedback loop that undermines antitumor efficacy. The compensatory hyperinsulinemia triggered by therapy can paradoxically reactivate mitogenic signaling (via IGF-1R) in residual tumor cells, thereby fostering resistance (39). Consequently, managing CTIM through lifestyle interventions, such as dietary modulation and exercise, is identified not merely as supportive care, but as an integral strategy to reverse metabolic reprogramming and improve long-term survival (17).

#### 4.2.3 Hotspot 3: The implications of dysregulated glucose metabolism and insulin resistance for cancer progression and treatment

A substantial body of evidence indicates that abnormal glucose metabolism and insulin resistance constitute a major biological conduit linking MetS to increased cancer incidence, accelerated tumor progression, and inferior clinical outcomes. Large-scale cohort studies indicate that type 2 diabetes confers an elevated risk for malignancies even after adjusting for confounders (41, 42). The central mechanistic driver is compensatory hyperinsulinemia rather than hyperglycemia alone. Insulin and IGF-1 signaling not only stimulate proliferation via the PI3K/AKT axis but also facilitate metabolic reprogramming toward lipogenesis and glycolysis (43). Consistent with this, the bioinformatic analysis identified AKT1 as a central hub gene, further supporting the importance of insulin and nutrient-sensing pathways in the MetS-cancer link. These endocrine alterations provide tumor cells with selective advantages, enhancing survival under oxidative and therapeutic stress (41). Integrative bioinformatics and multi−omics analyses further link aberrant insulin/IGF−1 signaling to metabolic heterogeneity in cancer, supporting that metabolic profiling can identify distinct metabolic subtypes associated with treatment resistance and prognosis (44).

Inhibition of insulin and glucose metabolism has potential in cancer treatment. The study showed that insulin-lowering dietary interventions, such as ketogenic diets, fasting-mimicking regimens, and caloric restriction, can effectively suppress circulating insulin and dampen tumor glucose availability (45). High insulin levels often drive resistance to targeted therapies by reactivating upstream signaling pathways; therefore, lowering systemic insulin disrupts this metabolic plasticity (46). Such interventions have been shown to sensitize tumors to cytotoxic and endocrine treatments, establishing a potent adjunctive avenue for managing metastatic disease (45). Insulin-lowering interventions offer a promising adjunctive therapeutic avenue that merits rigorous mechanistic and clinical investigation.

#### 4.2.4 Hotspot 4: lifestyle factors, obesity, and cancer risk

Diet, physical inactivity, and circadian disruption are among the most clinically actionable factors linking metabolic dysregulation to carcinogenesis. Studies revealed that transitioning to a hypercaloric, sedentary state triggers a systemic pro-inflammatory phenotype. This milieu is characterized by altered adipokine secretion and insulin resistance, which activate oncogenic pathways such as the IL-6/STAT3 and NF-κB across multiple malignancies (47, 48). Crucially, this pathology constitutes an ordinary soil for both cancer and cardiovascular disease (CVD). These comorbidities share overlapping etiologies rooted in chronic oxidative stress and endothelial dysfunction (49). Thus, risk factors like dyslipidemia mechanistically fuel tumorigenesis through shared inflammatory cascades. This understanding has catalyzed interest in chrononutrition. Interventions like time-restricted eating (TRE) align nutrient intake with circadian rhythms, thereby downregulating mTOR signaling and improving metabolic homeostasis (50, 51). Furthermore, specific populations exhibit heightened vulnerability to this axis. In a 2025 study, Corominas-Herrero et al. identified that childhood leukemia survivors face significant risks for early-onset MetS due to treatment-induced adipose dysfunction. Consequently, personalized lifestyle interventions are critical for mitigating secondary malignancies and cardiovascular events in these long-term survivors (52).

#### 4.2.5 Hotspot 5: Cancer cachexia as a distinct paraneoplastic MetS

Cancer cachexia, affecting approximately half of all cancer patients, represents a lethal MetS characterized by the progressive depletion of skeletal muscle and adipose tissue that cannot be fully reversed by nutritional support alone. This state was defined not merely as an energy deficit but as a systemic disruption of host metabolism driven by a negative protein and energy balance (53). Unlike simple starvation, cachexia is caused by malignant inflammation, which describes the complex interplay between tumor-derived factors such as ZAG, PTHrP and host cytokines such as TNF-α, IL-6. Recent integrative multi-omics studies highlight cancer metabolic heterogeneity, linking inflammatory mediators and oncogenic signaling such as STAT3, TP53 to systemic metabolic dysregulation (54). This metabolic dysregulation induces insulin resistance and triggers a hypermetabolic state characterized by futile energy cycling (55). At the molecular level, these inflammatory mediators suppress anabolic pathways (PI3K/Akt) while activating catabolic machinery. A hallmark mechanism is the upregulation of the ubiquitin-proteasome pathway via FoxO transcription factors, leading to accelerated muscle proteolysis (56, 57). Mechanistically, cancer cachexia shares distinct pathophysiological pathways with age-related sarcopenia and frailty, suggesting that preserving muscle mass is critical for maintaining organismal resilience against both cancer and ageing. Simultaneously, lipid metabolism is reprogrammed. Cachexia involves not only enhanced lipolysis but also the browning of white adipose tissue, which increases thermogenesis and energy expenditure. This coordinated catabolism of fat and lean mass severely compromises patient survival and tolerance to antineoplastic therapy, underscoring cachexia as a critical metabolic target rather than a passive side effect (58).

Crucially, these cachectic metabolic perturbations, like tumor-driven insulin resistance, systemic dyslipidemia, and chronic cytokine-mediated inflammation, recapitulate core metabolic features of metabolic syndrome, arising independently of any therapeutic intervention. Insulin resistance often manifests in cachectic patients even before significant weight loss, driven by tumor-secreted mediators that enhance hepatic gluconeogenesis and impair peripheral glucose uptake, rather than by treatment-related toxicity (59). Concurrently, tumor-derived factors including ZAG, PTHrP, and IL-6 orchestrate lipolysis and adipose tissue remodeling, producing dyslipidemia and hypermetabolism reminiscent of metabolic syndrome lipid disturbances (59). Together, these mechanisms provide direct evidence that cancer itself can induce *de novo* metabolic derangements beyond treatment-related effects through systemic secretory and inflammatory activity.

## 5 Limitations

This study has several limitations. Bibliometric analyses were limited to Web of Science and PubMed, which may introduce bias related to database coverage, language selection, and citation-based metrics, and the use of only these two databases may have resulted in incomplete retrieval of relevant studies from other sources such as Scopus or Embase. In the bioinformatic analysis, the GeneCards relevance score threshold and the high STRING confidence cutoff were used to improve the specificity and reliability of the identified targets and interactions; in particular, the 0.9 threshold allowed retention of high-confidence PPI data with strong biological relevance, although this stringent criterion may also have excluded some potentially relevant but less-characterized proteins and reduced network density. The identified hub genes and enriched pathways were derived from existing databases and network models and were not independently validated in the present study, although related validation has been reported in previous studies. In addition, heterogeneity in MetS definitions, population-specific thresholds, cancer types, study design, and follow-up duration limits cross-study comparability and generalizability. While this review extends discussion of the cancer-to-MetS direction beyond treatment-related effects, this aspect of the MetS-cancer relationship remains less extensively characterized than the MetS-to-cancer pathway and requires further substantiation. Finally, the absence of multi-omics integration and longitudinal patient-level data restricts mechanistic resolution. Future studies should incorporate large-scale prospective cohorts and multidimensional datasets to better define the dynamic MetS-cancer axis.

## 6 Conclusion

This study conducted a bibliometric and bioinformatics analysis of research on metabolic syndrome and cancer from 2000 to 2024, quantifying productivity and collaboration patterns at the national, institutional, and individual levels, and elucidating key findings and hotspots in the research. By integrating large-scale bibliometric mining, molecular network analysis, and clinical trial evidence, the study provides convergent and data-driven support for a robust, bidirectional connection between metabolic syndrome and cancer. Beyond reinforcing epidemiological associations, our findings highlight shared regulatory hubs and signaling pathways that mechanistically couple host metabolic dysregulation to tumor initiation, progression, and therapeutic response. Our findings substantiate that MetS and cancer are not merely comorbid conditions, but rather share overlapping molecular pathways, including chronic inflammation, dysregulated insulin signaling, and oncogenic transcriptional activation that mechanistically couple host metabolic dysregulation to tumor initiation and progression. Collectively, these observations suggest a conceptual shift in oncology, from a tumor-centric model to a holistic management of the tumor–host metabolic ecosystem. Future cancer research and treatment strategies should move beyond cytotoxicity alone and prioritize metabolic modulation as a complementary avenue to improve prognosis, treatment tolerance, and long-term survival, thereby opening new opportunities for precision prevention and metabolism-oriented oncologic interventions.

## Data Availability

The original contributions presented in the study are included in the article/**Supplementary Material**. Further inquiries can be directed to the corresponding authors.
