# Supplementary material for: The metabolic syndrome-cancer axis: global research trends and clinical landscapes
Source: Front Endocrinol (Lausanne). 2026 Jun 22;17:1799202. doi: 10.3389/fendo.2026.1799202 (PMC13343530; doi:10.3389/fendo.2026.1799202)
Supplement: Supplementary file 1 [file DataSheet1.docx]

**Identification of studies via Web of Science Core Collection (WoSCC) and ClinicalTrials.gov**

WoSCC Records removed *before screening*:

Duplicate records removed (n = 3)

Retracted or incomplete records (n = 2)

ClinicalTrials.gov Records removed *before screening*: (n = 0)

Records identified from*:

WoSCC (n = 714)

ClinicalTrials.gov (n = 25)

**Identification**

Records excluded through title**

WoSCC

Not related to MetS (n = 10)

Not related to cancer (n = 4)

ClinicalTrials.gov

Not related to MetS (n = 3)

Not related to cancer (n = 1)

Records screened

WoSCC (n = 709)

ClinicalTrials.gov (n = 25)

Reports not retrieved

WoSCC (n = 2)

ClinicalTrials.gov (n = 0)

Reports sought for retrieval

WoSCC (n = 695)

ClinicalTrials.gov (n = 21)

**Screening**

Reports excluded through abstract:

WoSCC

Not related to MetS (n =11)

Not related to cancer (n = 6)

ClinicalTrials.gov

Not related to MetS (n = 2)

Not related to cancer (n = 2)

Reports assessed for eligibility

WoSCC (n = 693)

ClinicalTrials.gov (n = 24)

Studies included in bibliometric analysis (n = 676)

Studies included in clinical trial analysis (n = 17)

**Included**

*Consider, if feasible to do so, reporting the number of records identified from each database or register searched (rather than the total number across all databases/registers).

**If automation tools were used, indicate how many records were excluded by a human and how many were excluded by automation tools.

Source: Page MJ, et al. BMJ 2021;372:n71. doi: 10.1136/bmj.n71.

This work is licensed under CC BY 4.0. To view a copy of this license, visit <https://creativecommons.org/licenses/by/4.0/>
